# Multi‐ancestry genome‐wide association study of asthma exacerbations

**DOI:** 10.1111/pai.13802

**Published:** 2022-06-08

**Authors:** Esther Herrera‐Luis, Victor E. Ortega, Elizabeth J. Ampleford, Yang Yie Sio, Raquel Granell, Emmely de Roos, Natalie Terzikhan, Ernesto Elorduy Vergara, Natalia Hernandez‐Pacheco, Javier Perez‐Garcia, Elena Martin‐Gonzalez, Fabian Lorenzo‐Diaz, Simone Hashimoto, Paul Brinkman, U‐BIOPRED Study Group, Andrea L. Jorgensen, Qi Yan, Erick Forno, Susanne J. Vijverberg, Ryan Lethem, Antonio Espuela‐Ortiz, Mario Gorenjak, Celeste Eng, Ruperto González‐Pérez, José M. Hernández‐Pérez, Paloma Poza‐Guedes, Olaia Sardón, Paula Corcuera, Greg A. Hawkins, Annalisa Marsico, Thomas Bahmer, Klaus F. Rabe, Gesine Hansen, Matthias Volkmar Kopp, Raimon Rios, Maria Jesus Cruz, Francisco‐Javier González‐Barcala, José María Olaguibel, Vicente Plaza, Santiago Quirce, Glorisa Canino, Michelle Cloutier, Victoria del Pozo, Jose R. Rodriguez‐Santana, Javier Korta‐Murua, Jesús Villar, Uroš Potočnik, Camila Figueiredo, Michael Kabesch, Somnath Mukhopadhyay, Munir Pirmohamed, Daniel B. Hawcutt, Erik Melén, Colin N. Palmer, Steve Turner, Anke H. Maitland‐van der Zee, Erika von Mutius, Juan C. Celedón, Guy Brusselle, Fook Tim Chew, Eugene Bleecker, Deborah Meyers, Esteban G. Burchard, Maria Pino‐Yanes

**Affiliations:** ^1^ Genomics and Health Group Department of Biochemistry, Microbiology, Cell Biology and Genetics Universidad de La Laguna (ULL) San Cristóbal de La Laguna, Tenerife Spain; ^2^ Division of Respiratory Medicine Department of Internal Medicine Mayo Clinic Scottsdale Arizona USA; ^3^ Department of Internal Medicine Center for Precision Medicine Wake Forest School of Medicine Winston‐Salem North Carolina USA; ^4^ 37580 Department of Biological Sciences National University of Singapore Singapore City Singapore; ^5^ MRC Integrative Epidemiology Unit (IEU) Population Health Sciences Bristol Medical School University of Bristol Bristol UK; ^6^ Department of Epidemiology Erasmus University Medical Center Rotterdam The Netherlands; ^7^ Department of Respiratory Medicine Ghent University Hospital Ghent Belgium; ^8^ Institute of Computation Biology Helmholtz Zentrum München German Research Center for Environmental Health Munich Germany; ^9^ Department of Clinical Sciences and Education Södersjukhuset Karolinska Institutet Stockholm Sweden; ^10^ CIBER de Enfermedades Respiratorias (CIBERES) Madrid Spain; ^11^ Instituto Universitario de Enfermedades Tropicales y Salud Pública de Canarias (IUETSPC) Universidad de La Laguna (ULL) San Cristóbal de La Laguna, Tenerife Spain; ^12^ Department of Respiratory Medicine Amsterdam University Medical Center University of Amsterdam Amsterdam The Netherlands; ^13^ 4591 Department of Health Data Science Institute of Population Health University of Liverpool Liverpool UK; ^14^ Department of Obstetrics and Gynecology Columbia University Irving Medical Center New York New York USA; ^15^ Division of Pediatric Pulmonary Medicine UPMC Children's Hospital of Pittsburgh University of Pittsburgh Pittsburgh Pennsylvania USA; ^16^ Department of Respiratory Medicine Amsterdam UMC University of Amsterdam Amsterdam The Netherlands; ^17^ Division of Pharmacoepidemiology and Clinical Pharmacology Faculty of Science Utrecht University Utrecht The Netherlands; ^18^ Department of Paediatric Respiratory Medicine and Allergy Emma's Children Hospital Amsterdam UMC University of Amsterdam Amsterdam The Netherlands; ^19^ Center for Human Molecular Genetics and Pharmacogenomics Faculty of Medicine University of Maribor Maribor Slovenia; ^20^ Department of Medicine University of California San Francisco San Francisco California USA; ^21^ Allergy Department Hospital Universitario de Canarias Santa Cruz de Tenerife Tenerife Spain; ^22^ Severe Asthma Unit, Allergy Department Hospital Universitario de Canarias Santa Cruz de Tenerife Tenerife Spain; ^23^ Pulmonary Medicine Hospital Universitario de N.S de Candelaria Santa Cruz de Tenerife Spain; ^24^ Pulmonary Medicine Hospital General de La Palma La Palma, Santa Cruz de Tenerife Spain; ^25^ Division of Pediatric Respiratory Medicine Hospital Universitario Donostia San Sebastián Spain; ^26^ Department of Pediatrics University of the Basque Country (UPV/EHU) San Sebastián Spain; ^27^ Department of Biochemistry Wake Forest School of Medicine Winston‐Salem North Carolina USA; ^28^ Computational Health Center Helmholtz Zentrum München German Research Center for Environmental Health Munich Germany; ^29^ LungenClinic Grosshansdorf, Pneumology Grosshansdorf Germany; ^30^ Airway Research Center North (ARCN) Members of the Germany Center for Lung Research (DZL) Grosshansdorf Germany; ^31^ Department of Pediatric Pneumology, Allergology and Neonatology Hannover Medical School Hannover Germany; ^32^ Division of Pediatric Pneumology & Allergology University Medical Center Schleswig‐Holstein Lübeck Germany; ^33^ Airway Research Center North (ARCN) Members of the Germany Center for Lung Research (DZL) Lübeck Germany; ^34^ Department of Paediatric Respiratory Medicine Inselspital University Children's Hospital of Bern University of Bern Bern Switzerland; ^35^ Programa de Pós Graduação em Imunologia (PPGIm) Instituto de Ciências da Saúde Universidade Federal da Bahia (UFBA) Salvador Brazil; ^36^ Servicio de Neumología Hospital Vall d’Hebron Barcelona Spain; ^37^ Servicio de Neumología Complejo Hospitalario Universitario de Santiago Santiago de Compostela Spain; ^38^ Servicio de Alergología Complejo Hospitalario de Navarra Pamplona Navarra Spain; ^39^ Departamento de Medicina Respiratoria Hospital de la Santa Creu i Sant Pau Instituto de Investigación Biomédica Sant Pau (IIB Sant Pau) Barcelona Spain; ^40^ Department of Allergy La Paz University Hospital IdiPAZ Madrid Spain; ^41^ Behavioral Sciences Research Institute University of Puerto Rico San Juan Puerto Rico; ^42^ Department of Pediatrics University of Connecticut Farmington Connecticut USA; ^43^ Immunology Department Instituto de Investigación Sanitaria Hospital Universitario Fundación Jiménez Díaz Madrid Spain; ^44^ Centro de Neumología Pediátrica San Juan Puerto Rico; ^45^ Multidisciplinary Organ Dysfunction Evaluation Research Network Research Unit Hospital Universitario Dr. Negrín Las Palmas de Gran Canaria Spain; ^46^ Laboratory for Biochemistry, Molecular Biology and Genomics Faculty for Chemistry and Chemical Engineering University of Maribor Maribor Slovenia; ^47^ Instituto de Ciências da Saúde Universidade Federal da Bahia Salvador Brazil; ^48^ Department of Paediatric Pneumology and Allergy University Children's Hospital Regensburg (KUNO) Regensburg Germany; ^49^ Academic Department of Paediatrics Brighton and Sussex Medical School, Royal Alexandra Children's Hospital Brighton UK; ^50^ 3042 Population Pharmacogenetics Group Biomedical Research Institute Ninewells Hospital and Medical School University of Dundee Dundee UK; ^51^ 4591 Department of Pharmacology and Therapeutics Institute of Systems, Molecular and Integrative Biology University of Liverpool Liverpool UK; ^52^ 4591 Department of Women's and Children's Health University of Liverpool Liverpool UK; ^53^ Alder Hey Children's Hospital Liverpool UK; ^54^ NIHR Alder Hey Clinical Research Facility Alder Hey Children's Hospital Liverpool UK; ^55^ Sachs’ Children’s Hospital South General Hospital Stockholm Sweden; ^56^ 1019 Child Health University of Aberdeen Aberdeen UK; ^57^ Institute for Asthma and Allergy Prevention Helmholtz Zentrum München German Research Center for Environmental Health Munich Germany; ^58^ Dr von Hauner Children's Hospital Ludwig‐Maximilians‐Universität Munich Germany; ^59^ Comprehensive Pneumology Center Munich (CPC‐M) Member of the German Center for Lung Research Munich Germany; ^60^ Department of Respiratory Medicine Erasmus University Medical Center Rotterdam The Netherlands; ^61^ Division of Genetics, Genomics, and Precision Medicine Department of Internal Medicine University of Arizona College of Medicine Tucson Arizona USA; ^62^ Department of Bioengineering and Therapeutic Sciences University of California San Francisco San Francisco California USA; ^63^ Instituto de Tecnologías Biomédicas (ITB) Universidad de La Laguna (ULL) San Cristóbal de La Laguna, Tenerife Spain

**Keywords:** asthma exacerbations, *EXTL2*, GWAS, *PANK1*, single‐nucleotide polymorphism

## Abstract

**Background:**

Asthma exacerbations are a serious public health concern due to high healthcare resource utilization, work/school productivity loss, impact on quality of life, and risk of mortality. The genetic basis of asthma exacerbations has been studied in several populations, but no prior study has performed a multi‐ancestry meta‐analysis of genome‐wide association studies (meta‐GWAS) for this trait. We aimed to identify common genetic loci associated with asthma exacerbations across diverse populations and to assess their functional role in regulating DNA methylation and gene expression.

**Methods:**

A meta‐GWAS of asthma exacerbations in 4989 Europeans, 2181 Hispanics/Latinos, 1250 Singaporean Chinese, and 972 African Americans analyzed 9.6 million genetic variants. Suggestively associated variants (*p* ≤ 5 × 10^−5^) were assessed for replication in 36,477 European and 1078 non‐European asthma patients. Functional effects on DNA methylation were assessed in 595 Hispanic/Latino and African American asthma patients and in publicly available databases. The effect on gene expression was evaluated in silico.

**Results:**

One hundred and twenty‐six independent variants were suggestively associated with asthma exacerbations in the discovery phase. Two variants independently replicated: rs12091010 located at vascular cell adhesion molecule‐1/exostosin like glycosyltransferase‐2 (*VCAM1*/*EXTL2*) (discovery: odds ratio (OR_T allele_) = 0.82, *p* = 9.05 × 10^−6^ and replication: OR_T allele_ = 0.89, *p* = 5.35 × 10^−3^) and rs943126 from pantothenate kinase 1 (*PANK1*) (discovery: OR_C allele_ = 0.85, *p* = 3.10 × 10^−5^ and replication: OR_C allele_ = 0.89, *p* = 1.30 × 10^−2^). Both variants regulate gene expression of genes where they locate and DNA methylation levels of nearby genes in whole blood.

**Conclusions:**

This multi‐ancestry study revealed novel suggestive regulatory loci for asthma exacerbations located in genomic regions participating in inflammation and host defense.

**Abbreviations**
1KGP: 1000 Genomes Project
CDK6: cyclin‐dependent kinase 6
CI: confidence interval
GAG: glycosaminoglycan
GTEx: genotype‐tissue expression
GWAS: genome‐wide association study
LPS: lipopolysaccharide
MAF: minor allele frequency
meQTL: methylation quantitative trait loci
OR: odds ratio
PPAR‐α: peroxisome proliferator‐activating receptor α
RR: relative risk
SNP: single‐nucleotide polymorphism
TLR4: Toll‐like receptor 4
TNFα: tumor necrosis factor α

**Key Message**

A large multi‐ancestry meta‐analysis of GWAS of asthma exacerbations revealed two novel susceptibility loci located close to *PANK1* and at the intergenic region of *VCAM1* and *EXTL2*. These loci decreased *PANK1* and *EXTL2* gene expression in whole blood, respectively. Both genetic variants were associated with DNA methylation levels at CpG sites nearby. Our results identified two gene targets for asthma exacerbations that should be further explored to assess their specific role in asthma.

## 1 INTRODUCTION

Asthma is a common chronic inflammatory airway disorder affecting over 300 million people worldwide. The disparities in asthma prevalence across populations reflect a complex interplay between environmental exposures (i.e., air pollution and viral infections), behavioral and socioeconomic factors (i.e., treatment adherence and healthcare access), and genetic ancestry, which is inferred from whole‐genome variation and tracks geographic and historical factors and the aforementioned factors influencing asthma prevalence.^1^, ^2^

Asthma exacerbations are defined as worsening of respiratory symptoms requiring hospitalization, unscheduled/emergency asthma care, and/or use of systemic corticosteroids.^3^ Prevention of asthma exacerbations is a major public health priority due to their associated consequences on health (i.e., decreased quality of life, accelerated decline in lung function, or mortality), school attendance, work productivity, and healthcare costs.^1^, ^4^, ^5^ To date, the best predictor of future exacerbations is the occurrence of one in the previous year.^6^ Thus, identifying potential biomarkers to guide the reduction and prevention of exacerbations is a priority for therapeutics development and for precision medicine of asthma.

With the advent of high‐throughput sequencing and genotyping technologies, the study of the genetic contributions to asthma exacerbations has shifted from hypothesis‐driven, limited candidate‐gene strategies to genome‐wide association studies (GWAS).^7^, ^8^, ^9^, ^10^, ^11^, ^12^, ^13^, ^14^ Pharmacogenomics studies of asthma exacerbations as an outcome of treatment response have identified five suggestive associations for asthma exacerbations despite inhaled corticosteroids (*CMTR1*,^9^ *APOBEC3B*‐*APOBEC3C*,^8^ and *CACNA2D3*‐*WNT5A* ^11^), or long‐acting beta2‐agonists (*TBX3* and *EPHA7*).^10^ Beyond pharmacogenomics, other studies have focused on asthma exacerbations independently of treatment. In European‐descent populations, *CDHR3*, *CTNNA3*, and *HLA*‐*DQB1* have been associated with severe asthma exacerbations.^7^, ^13^ More recently, the representation of ethnically diverse populations has increased in GWAS of asthma exacerbations. A meta‐analysis of GWAS in Hispanic/Latino children identified a single‐nucleotide polymorphism (SNP) at *FLJ22447* that modulated *KCNJ2*‐*AS1* expression in nasal epithelium through DNA methylation.^12^ In Hispanic/Latinos and African Americans, a genome‐wide significant locus for asthma with exacerbations regulated *LINC01913* lung gene expression and DNA methylation levels of the *PKDCC* gene in whole blood.^14^ However, none of those studies has approached the search for genetic determinants of asthma exacerbations independently of treatment from a multi‐ancestry framework.

To improve our understanding on genetic and biological mechanisms of asthma exacerbations across multiple populations, we conducted the first multi‐ancestry meta‐analysis of GWAS of asthma exacerbations independently of treatment and attempted to validate previous associations. Then, we conducted *in silico* and *in vivo* downstream analyses to assess the potential functional effects of the associated SNPs over DNA methylation and gene expression.

## 2 METHODS

### 2.1 Study design and study populations

We performed a two‐stage study to identify genetic variants associated with asthma exacerbations, defined as a binary variable based on the presence of emergency care, hospitalizations, or administration of systemic corticosteroids because of asthma. We also considered a definition of moderate exacerbations,^3^ comprising unscheduled general practitioner or pulmonary specialist visits and school absence, as no information on the former variables was available for some studies. A period of 6–24 months or ever was considered depending on the data available for each study (Tables S1 and S2). In the discovery phase, we performed a multi‐ancestry meta‐analysis of GWAS of asthma exacerbations in 9392 patients with asthma from 12 studies, including 4989 European‐descents from nine studies, 2181 Hispanics/Latinos, 1250 Singaporean Chinese, and 972 African Americans. We attempted to replicate the findings from the discovery phase in a total of 37,555 participants with asthma, including 36,477 Europeans from seven studies, 877 Latinos from two studies, and 201 Filipinos from one study (Table S2). A detailed description of each study is available in the Appendix S1. All studies included were approved by their respective Institutional review boards, and written informed consent was provided by participants or their parents/caregivers. All methods followed the Declaration of Helsinki guidelines.

Assessment of genetic ancestry was performed using principal component analysis. The Haplotype Reference Consortium (r1.1 2016)^15^ was used as the reference imputation panel for most studies, except for Avon Longitudinal Study of Parents and Children (ALSPAC) and Singapore Cross Sectional Genetic Epidemiology Study (SCSGES), which used the phase 3 of the 1000 Genomes Project (1KGP).^16^ Genotyping and imputation procedures for the discovery and replication studies are detailed in the Appendix S1 and Tables S1 and S2.

### 2.2 Association analysis

Association between genetic variants and asthma exacerbations was tested using logistic regression models including age, sex, and principal components from the genotype matrix (if needed to correct for population stratification) (Table S1). Analyses were conducted separately for each study using PLINK 2.0,^17^ EPACTS 3.2.6^18^ or rvtests 2.1.0.^19^ Results were filtered with the EasyQC software^20^ to retain variants with a minor allele frequency (MAF) ≥ 1% and imputation quality *R* ^2^ ≥ .3, absolute value of the beta coefficient <10, standard error of the beta included in the interval [0,10], and minor allele cut‐off ≥6.

In the discovery phase, genetic variants that were available in at least two ethnic‐specific studies were meta‐analyzed with METASOFT,^21^ using fixed‐effects or random‐effects models based on the heterogeneity among studies (measured by the Cochran’s *Q* test *p*‐value). Ethnic‐specific results were then combined in a multi‐ancestry meta‐analysis. Independent variants (*r* ^2^ ≤ .8) with suggestive association^22^ at *p* ≤ 5 × 10^−5^ within 1 Megabase were identified with GCTA‐COJO v1.93.2^23^ using the 1KGP reference.^16^ These variants were evaluated in the replication stage, following the same procedures as in the discovery phase. Evidence of replication was considered if the variants showed consistent direction of effects with the discovery stage at *p* ≤ .05.

### 2.3 Assessment of shared genetic basis of asthma exacerbations with other traits

To identify groups of genes previously associated with other traits, we used a Gene‐Set Enrichment Analysis (GSEA), as implemented in FUMA GWAS^24^ via the *GENE2FUNC* algorithm, and queried the GWAS catalog.^25^ SNPs with *p* ≤ 1 × 10^−4^ in the discovery phase of the meta‐analysis of GWAS were mapped to the closest gene using the UCSC Table Browser tool.^26^ A false discovery rate (FDR) of 5% was used to declare significance.

To estimate the pairwise genome‐wide genetic correlations (*R*_g_) between asthma exacerbations and other traits, we compared our findings with publicly available GWAS summary statistics via LD score regression using LDHub.^27^ As most of the GWAS have been conducted in European populations, the analysis was restricted to predominantly European‐descent individuals to maximize the statistical power. A Bonferroni‐corrected significance threshold of *p* < .05/711 traits = 6.48 × 10^−5^ was applied.

### 2.4 Sensitivity analysis

To assess the robustness of the genetic associations, we conducted sensitivity analyses for the time‐dependent probability occurrence of exacerbations, the effect of Body Mass Index (BMI), obesity, asthma severity, and age group. Moreover, we evaluated the association of the variants with asthma susceptibility, as detailed in the Appendix S1. Studies from the discovery stage that had covariate data available were considered.

### 2.5 Methylation profiling and quality control

Whole blood DNA methylation from Hispanics/Latinos and African Americans was profiled using the Infinium HumanMethylation450 BeadChip or the Infinium Methylation EPIC BeadChip arrays. Briefly, low‐quality probes and samples, outliers of DNA methylation, and samples with sex mismatch or mixed genotype distributions on the control SNP probes were excluded. Standard background correction, dye‐bias correction, inter‐array normalization, and probe‐type bias adjustment were performed, and beta values were transformed to *M*‐values for better statistical performance. Quality control is detailed in the Appendix S1.

### 2.6 Functional assessment of associated SNPs

DNA methylation quantitative trait loci (meQTL) analyses were conducted using fastQTL^28^ for CpG sites within 1 Mb of SNPs with MAF ≥ 0.01 in at least 10 samples, separately in 139 Mexican Americans and 241 Puerto Ricans from Genes‐Environments & Admixture in Latino Americans (GALA II) and 215 African Americans from the Study of African Americans, Asthma, Genes & Environments (SAGE) studies. Linear regression models were corrected for asthma exacerbations status, age, sex, genetic ancestry, ReFACTor components as a proxy of cell heterogeneity, and methylation batch (when appropriate). The results from Mexican Americans and Puerto Ricans assayed with different methylation arrays were then meta‐analyzed for each sub‐ethnic group with METASOFT.^21^ SNP‐CpG pairs were considered significant at Storey *q*‐value <.05. In silico evidence of functional effects of variants on gene expression and DNA methylation was assessed using QTLbase,^29^ Genotype‐Tissue Expression (GTEx) v8 Portal,^30^ PhenoScanner v2^31^ and eFORGE‐TF.^32^ Long‐distance chromatin interactions were determined using the ChiCP tool.^33^

### 2.7 Validation of previous associations

A literature search for all studies reporting genetic loci significantly associated with asthma exacerbations was conducted, as described in the Appendix S1. Association results in the discovery stage were extracted and significance threshold was defined as *p* = .05/ number of tested SNPs to adjust for multiple testing.

## 3 RESULTS

### 3.1 Characteristics of the patients

In the discovery phase, we analyzed 2781 exacerbators and 6611 non‐exacerbators; 53.1% were predominantly Europeans, 23.2% Hispanics/Latinos, 13.3% Singaporean Chinese, and 10.3% African Americans. The percentage of exacerbators ranged from 9.1% to 65.2% in Europeans, and reached 58.8% in Hispanics/Latinos, 46.1% in African Americans, and 3.4% in Singaporeans. The replication phase included 37,555 individuals with asthma (3030 exacerbators and 34,525 non‐exacerbators) where most participants were of European descent (97.1%), followed by Latinos (2.3%) and Filipinos (0.5%). The percentage of exacerbators ranged from 4.8% to 65.2% in Europeans, reached approximately 43% in Latinos, and 1.3% in Filipinos (Tables S1 and S2). Regarding sex, 51.7% and 42.9% were male participants in the discovery and replication phases, respectively.

### 3.2 Discovery phase

**FIGURE 1:**
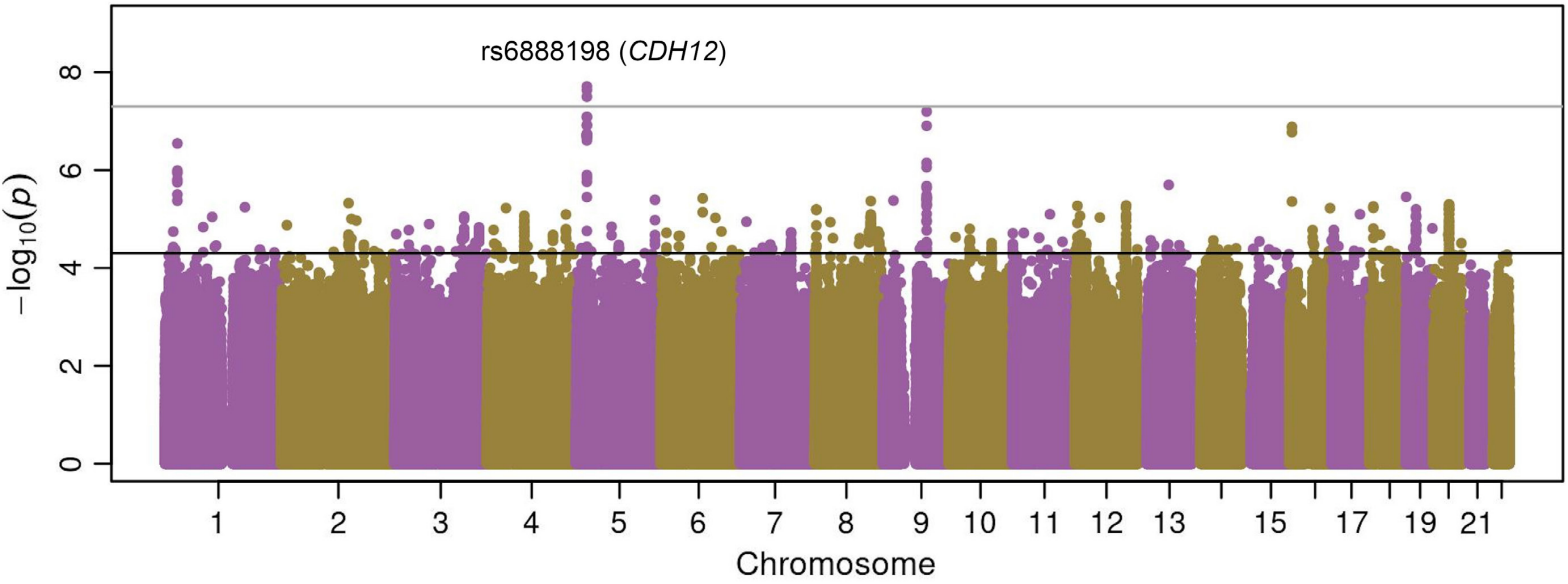
Manhattan plot of the results of the discovery stage of the multi‐ancestry meta‐analysis of GWAS of asthma exacerbations (represented as ‐log_10_ *p*‐value on the *y*‐axis) along the chromosome position of the variants analyzed (*x*‐axis). The suggestive (*p* = 5 × 10^−5^) and genome‐wide (*p* = 5 × 10^−8^) significance thresholds are indicated by the black line and dark gray lines

The quantile–quantile plots did not show major genomic inflation due to population stratification in each individual study (Figure S1), the combined results from individuals of European‐descent (Figure S2), or the multi‐ancestry meta‐analysis (Figure S3). In the multi‐ancestry meta‐analysis of 9,634,748 variants, 447 SNPs exhibited suggestive association (Table S3). The most significant association was the intronic SNP rs6888198 within the cadherin‐12 (*CDH12*) gene at chromosome 5p14.3 (odds ratio [OR] for C allele: 1.37, 95% confidence interval [CI]: 1.23–1.54, *p* = 1.95 × 10^−8^) (Figure 1, Figure S4).

### 3.3 Replication phase

**FIGURE 2:**
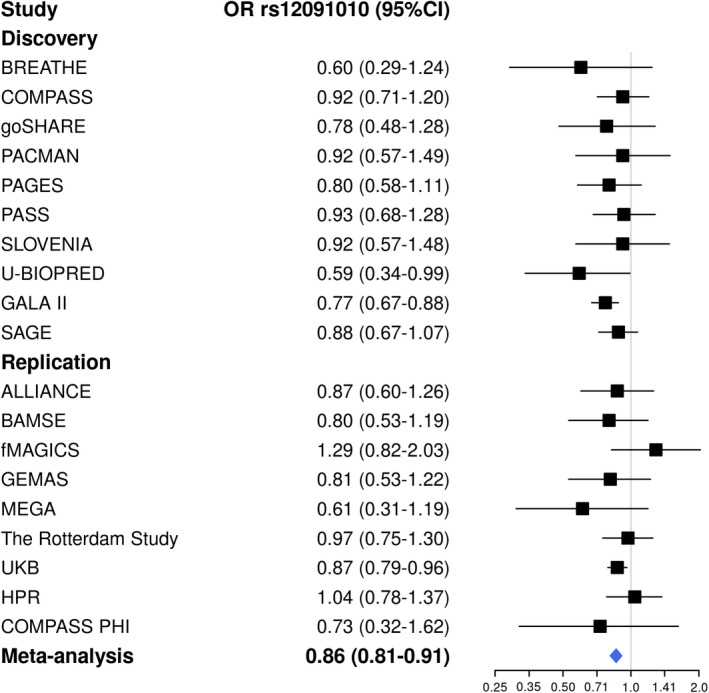
Forest plot of the association results for rs12091010 (*VCAM1*/*EXTL2*) in the meta‐analysis of GWAS of asthma exacerbations. ALSPAC (discovery), SCSGES (discovery), and the subset of samples from BREATHE genotyped with the Illumina Infinium CoreExome‐24 BeadChip (replication) had no genotyped or imputed data for rs12091010

**FIGURE 3:**
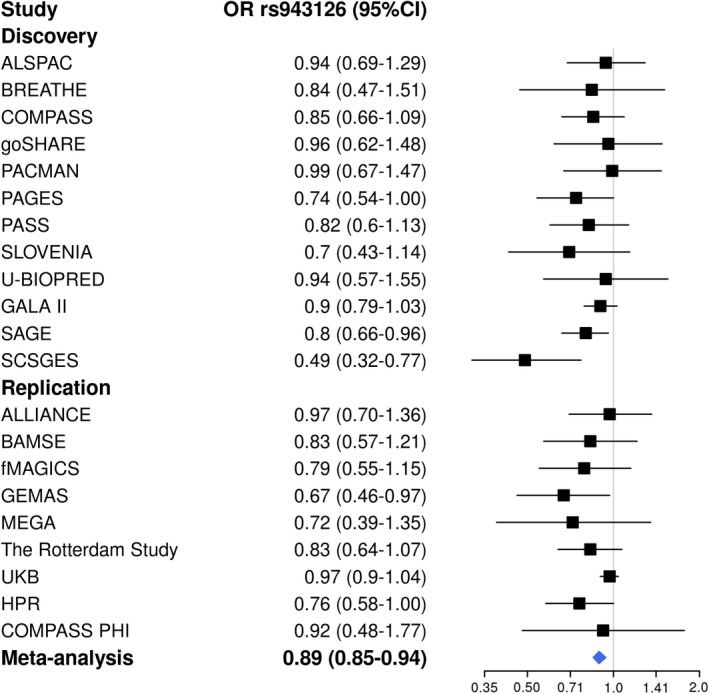
Forest plot of the association results for rs943126 (*PANK1*) in the meta‐analysis of GWAS of asthma exacerbations. The subset of samples from BREATHE genotyped with the Illumina Infinium CoreExome‐24 BeadChip (replication) had no available genotyped or imputed data for rs943126

Fifteen of the 126 independent variants identified in the discovery phase were not available for replication as they were mostly present in African Americans and Hispanics/Latinos (Table S3). Two of the 106 variants present in more than one ethnic group were consistently associated with asthma exacerbations (Table 1): rs12091010 [*VCAM1*/*EXTL2*, OR for T allele: 0.89 (0.82–0.97), *p* = 5.35 × 10^−3^] (Figure 2) and rs943126 [*PANK1*, OR for C allele: 0.92 (0.86–0.98), *p* = 1.30 × 10^−2^] (Figure 3). In the meta‐analysis across both phases, these variants reached an association *p*‐value of 4.23 × 10^−7^ and 4.93 × 10^−6^, respectively. From five variants that were present only in non‐Europeans in the replication stage, none exhibited *p* < .05 in any other population group (Table S4). Even though rs6888198 reached genome‐wide significance in the discovery and showed consistent effects among Europeans in the replication phase, this SNP had opposite effects in Latinos and Filipinos, which resulted in the lack of replication in the multi‐ancestry replication phase (Table 1, Figure S5).

**TABLE 1:** Association results for the top hit in the discovery stage and sentinel variants with significant and consistent effects in the discovery and replication phases, and the meta‐analysis across both phases

ID^a^	rsID	Closest gene	Discovery			Replication			Meta‐analysis (discovery and replication)		
			OR (95% CI)	*p*	Cochran's *Q p*	OR (95% CI)	*p*	Cochran's *Q p*	OR (95% CI)	*p*	Cochran's *Q p*
1:101210560:C:T	rs12091010	*EXTL2*	0.82 (0.75–0.90)	9.05E‐06	4.83E‐01	0.89 (0.82–0.97)	5.35E‐03	4.92E‐01	0.86 (0.81–0.91)	4.23E‐07	4.47E‐01
5:22659406:T:C	rs6888198	*CDH12*	1.37 (1.23–1.54)	1.95E‐08	5.82E‐01	1.02 (0.90–1.15)	7.72E‐01	7.18E‐01	1.24 (1.05–1.45)	2.41E‐06	1.89E‐02
10:91376299:T:C	rs943126	*PANK1*	0.85 (0.78–0.92)	3.10E‐05	8.01E‐02	0.92 (0.86–0.98)	1.30E‐02	3.87E‐01	0.89 (0.85–0.94)	4.93E‐06	7.91E‐02

Abbreviations: 95% CI, 95% confidence interval; OR, odds ratio; *p*, *p*‐value.

^a^ The variant identifier corresponds to chromosomal position (hg19) followed by non‐tested allele and tested allele.

### 3.4 Gene‐set enrichment and genome‐wide genetic correlation analysis

Enrichment analysis of associations from the multi‐ancestry discovery GWAS including 959 SNPs associated with asthma exacerbations at *p* ≤ 1 × 10^−4^ revealed significant enrichment in several traits, including treatment response (min *p* = 2.77 × 10^−6^), neurological conditions (min *p* = 4.62 × 10^−5^), obesity (min *p* = 6.52 × 10^−5^), or waist‐to‐hip ratio (min *p* = 1.88 × 10^−7^) (Table S5).

A total of 16 traits exhibited genetic correlation with asthma exacerbations at *p* < .05 (Table S6), including wheeze or whistling in the last year (*R*_g_ = 0.47, *p* = 1.01 × 10^−2^), emphysema/chronic bronchitis (*R*_g_ = 0.55, *p* = 3.89 × 10^−2^), asthma (*R*_g_ = 0.32, *p* = 3.99 × 10^−2^), and BMI (*R*_g_ = 0.19, *p* = 4.76 × 10^−2^). However, the associations did not remain significant after Bonferroni correction.

### 3.5 Sensitivity analysis

To assess the robustness of associations that replicated across stages to the time‐dependent probability of occurrence of exacerbations, stratified analyses were performed in European‐descents from the discovery stage that reported exacerbations for 6 vs. 12 months. Consistent effects per period were observed across periods (Table 2).

**TABLE 2:** Sensitivity analysis for rs12091010 and rs943126 in individuals from the discovery stage

	Exacerbations in the last 6 months			Exacerbations in the last 12 months					
	European‐descent populations			European‐descent populations			Multi‐ancestry meta‐analysis		
rsID	OR (95% CI)	*p*	Cochran's *Q p*	OR (95% CI)	*p*	Cochran's *Q p*	OR (95% CI)	*p*	Cochran's *Q p*
rs12091010	0.84 (0.67–1.04)	1.08 × 10^−1^	5.14 × 10^−1^	0.86 (0.72–1.03)	9.13 × 10^−2^	6.25 × 10^−1^	0.82 (0.74–0.90)	3.45 × 10^−5^	4.84 × 10^−1^
rs943126	0.78 (0.64–0.96)	2.13 × 10^−2^	8.61 × 10^−1^	0.88 (0.74–1.04)	1.26 × 10^−1^	8.28 × 10^−1^	0.85 (0.78–0.93)	3.29 × 10^−4^	7.50 × 10^−2^

Abbreviations: 95% CI, 95% confidence interval; OR, odds ratio; *p*, *p*‐value.

As the post‐GWAS analyses revealed significant enrichment/correlation at *p* < .05 with fat mass/distribution, the association of rs12091010 and rs943126 after additional adjustment by BMI/obesity was examined in individuals from the discovery phase with BMI data available. Moreover, the effect of asthma severity alone or combined with BMI/obesity on the genetic association exacerbations was evaluated. The effects sizes of the genetic association after additional adjustment by these variables remained consistent with the effects reported in the discovery stage (Table S7).

We next investigated if the observed effects could differ across age groups in those studies that analyzed exclusively children or adults, but the effect sizes remained consistent across age groups (Table S8). Moreover, we assessed if the effects could be driven by the underlying asthma syndrome, rather than asthma exacerbations, and no significant association with asthma was found in results from the UK Biobank or the Michigan Genomics Initiative (Table S9).

### 3.6 Functional exploration of variants associated with asthma exacerbations

We next assessed the association between DNA methylation levels in whole blood at 525 and 538 CpG sites with rs12091010 and rs943126, respectively. A total of 7 and 1 SNP‐CpG pairs for rs943126 and rs12091010 exhibited Storey *q* < .05, respectively (Table 3 and Table S10). Two of these replicated consistently in Europeans for rs943126 (cg25770176 and cg00475140). *In silico* analyses revealed 10 SNP‐CpGs pairs, 3 of which showed consistent effects in Hispanics/Latinos and African Americans at Storey *q* < .05 (Tables S11 and S12) including the previous two pairs and rs943126‐cg03948048. The eight significant CpG sites in minority children showed significant enrichment (*q* < .001) in transcription factor (TF) motifs in lung (Table S13). Besides, the T allele of rs12091010 was associated with decreased *EXTL2* expression in whole blood from Europeans, according to PhenoScanner.^31^ The C allele of rs943126 was associated with increased expression of *PANK1* in whole blood from Europeans (Table S14). Both variants showed evidence of long‐range chromatin interaction with several genes in lymphoblastoid cells, including *VCAM1* and *EXTL2* for rs12091010 and *PANK1* for rs943126 (Table S15).

**TABLE 3:** Results from the meQTL analysis in whole blood in the GALA II and SAGE studies for genome‐wide significant hit in the discovery and two SNPs that were replicated

SNP‐CpG pair	Position (hg19)	Closest genes	Mexican Americans			Puerto Ricans			African Americans			Meta‐analysis				
			Coef	SE	*p*	Coef	SE	*p*	Coef	SE	*p*	Coef	SE	*p*	Cochran's *Q p*	Storey *q*
rs943126‐cg26800131	91574784	*KIF20B*	−0.21	0.11	**2.99E‐04**	−0.07	0.04	6.99E‐02	−0.07	0.04	8.87E‐02	−0.18	0.10	**1.85E**‐**06**	4.45E−03	**9.95E**‐**04**
rs943126‐cg14920044	91296311	*SLC16A12*	0.09	0.05	9.77E‐02	0.24	0.06	**1.68E**‐**05**	0.12	0.06	**4.68E**‐**02**	0.15	0.03	**4.61E**‐**06**	1.24E‐01	**1.24E**‐**03**
rs943126‐cg20654695	91444521	*KIF20B/PANK1*	−0.10	0.08	2.12E‐01	−0.06	0.04	9.04E‐02	−0.15	0.03	**2.04E**‐**05**	−0.10	0.02	**9.96E**‐**06**	1.98E−01	**1.79E**‐**03**
rs943126‐cg25770176	91405685	*PANK1*	−0.09	0.03	**1.43E**‐**03**	0.00	0.09	2.78E‐01	−0.07	0.02	**3.00E**‐**03**	−0.07	0.02	**1.86E**‐**05**	1.49E−01	**2.50E**‐**03**
rs12091010‐cg05612904	101491636	*DPH5*	−0.07	0.04	6.99E‐02	−0.21	0.11	**2.99E**‐**04**	−0.09	0.04	**2.72E**‐**02**	−0.10	0.02	**2.31E**‐**05**	9.52E−02	**1.20E**‐**02**
rs943126‐cg00475140	91404454	*PANK1*	−0.21	0.07	**1.39E**‐**03**	−0.13	0.08	1.05E‐01	−0.19	0.09	**3.19E**‐**02**	−0.18	0.04	**4.28E**‐**05**	5.25E−01	**4.60E**‐**03**
rs943126‐cg15620114	91296457	*SLC16A12*	0.09	0.08	2.64E‐01	0.27	0.09	**3.87E**‐**03**	0.20	0.08	**7.76E**‐**03**	0.18	0.05	**1.53E**‐**04**	5.70E−01	**1.32E**‐**02**
rs943126‐cg04957662	91411382	*KIF20B/PANK1*	−0.34	0.79	6.69E‐01	−1.16	0.29	**1.33E**‐**04**	−0.77	0.26	**2.89E**‐**03**	−1.02	0.27	**1.72E**‐**04**	3.14E−01	**1.32E**‐**02**

Abbreviations: Coef, Coefficient of the regression; *p*, *p*‐value; SE, standard error; Storey *q*, Storey *q*‐value. Bold numbers corresponds to values less than .05 for both Storey *q* and *p*.

### 3.7 Validation of previous associations

We next examined 47 previous genetic loci for asthma exacerbations^7^, ^8^, ^12^, ^13^, ^34^, ^35^, ^36^ and moderate‐to‐severe asthma^37^ for association with asthma exacerbations in the discovery phase. A total of 5 variants had *p* < .05 in Europeans, 2 in Hispanics/Latinos, 5 in African Americans, and 1 in Singaporean Chinese (Table S16). These were in loci previously associated with asthma exacerbations (*GSDMB*, *RAD50*, *HLA*‐*DQB1*, *ADAM33*, *VDR*, and *CDHR3*) or moderate‐to‐severe asthma (*IKZF3*, *TSLP*, *MUC5AC*, *C11orf30*, *SMAD3*, and *WDR36*). However, none of the SNPs exceeded the stringent Bonferroni‐corrected threshold for significance (*p* = .05/47 = 1.06 × 10^−3^).

## 4 DISCUSSION

To our knowledge, this is the first multi‐ancestry meta‐analysis of GWAS of asthma exacerbations independently of treatment including European, Hispanic/Latino, Asian, and African American patients with asthma. In our combined analysis of 46,947 individuals with asthma, two regulatory SNPs were significantly and consistently associated with asthma exacerbations in most of the studies included in the discovery and replication phases, independently of the type of exacerbation and the time period for which the exacerbation status was assessed. The SNP rs120910109 was located in the intergenic region of the *VCAM1*/*EXTL2* genes, whereas rs943126 was harbored within intron 1 of *PANK1*.

*VCAM1* encodes a surface protein predominantly expressed in endothelial cells that modulates leukocyte adhesion and trans‐endothelial migration in response to pro‐inflammatory cytokines, and lipopolysaccharide (LPS) among other factors.^38^, ^39^ VCAM1 is involved in cancer progression and several immunological disorders, including asthma.^38^ In the ovalbumin mice model, anti‐VCAM1 reduced airway hyperresponsiveness and eosinophilic inflammation.^40^ On the other hand, *EXTL2* encodes an enzyme that controls glycosaminoglycan (GAG) biosynthesis via transference of N‐acetylgalactosamine and N‐acetylglucosamine to the glycosaminoglycan‐protein linkage region.^41^ Decreased *EXTL2* causes an over‐accumulation of GAGs^42^ that can promote inflammation in injured areas.^43^, ^44^ Moreover, in bone marrow‐derived macrophages from *EXTL2* ^−/−^ mice, there is overproduction of key molecules involved in inflammation and extracellular matrix remodeling, including tumor necrosis factor α (TNFα) and several matrix metalloproteinases.^43^ In a scenario of overaccumulation of GAGs under the loss of *EXTL2* in macrophages, GAGs act as inflammatory mediators with strong Toll‐like receptor 4 (TLR4) agonist capacity.^44^ Interestingly, genetic variation in both *VCAM1* and *EXTL2* is associated with blood cell counts and multiple sclerosis, according to the GWAS catalog.^25^

PANK1 catalyzes coenzyme A biosynthesis, regulated by the transcription factor peroxisome proliferator‐activating receptor α (PPAR‐α),^45^ a key anti‐inflammatory factor in asthma.^46^ A decrease in PPAR‐α expression is accompanied by a decrease in the expression of PANK1 and miR‐107, which is encoded within the intron 5 of *PANK1*. TLR4 can also downregulate miR‐107. In turn, this leads to a higher cyclin‐dependent kinase 6 (CDK6) expression and subsequently increases the adhesion of macrophages in response to LPS.^45^ Bioproducts from bacterial infections, such as LPS, can trigger an inflammatory response and increase airway hyperresponsiveness and risk of asthma exacerbations.^47^, ^48^ Moreover, p53 can regulate cell cycle progression via upregulation of PANK1 after DNA damage^49^ and metabolism.^50^

To prioritize gene targets, we assessed the functional capacity of relevant SNPs.^51^ Both rs12091010 and rs943126 exhibited an association with DNA methylation at several nearby CpG sites in whole blood from African Americans and Hispanics/Latinos with asthma. Additionally, the SNPs rs12091010 and rs943126 were associated with *EXTL2* and *PANK1* gene expression in whole blood from Europeans. Specifically, the T allele of rs12091010, located at 6 kb downstream of the 3′ UTR of *VCAM1* and 150 kb upstream of the transcription start site of *EXTL2*, was associated with lower odds of having asthma exacerbations and decreased *EXTL2* expression^31^ The T allele is more common among Latinos/Admixed Americans, followed by Europeans, Africans, and East Asians (Figure S6). The T allele of rs943126 at *PANK1*, which is less common among Europeans than the rest of populations (Figure S7), was associated with a higher risk of asthma exacerbations in the combined analysis of the discovery and replication phases and with decreased gene expression of *PANK1* in whole blood from Europeans according to PhenoScanner.^31^ However, these eQTL effects were not validated in the GTEx data.^30^

In the discovery phase, the most significant association was located at the intronic SNP rs6888198 (*CDH12)*, but no evidence of replication was found in the second stage (*p* > .05) despite the consistency of the direction of the effect across study phases. Interestingly, rs6888198 showed variable MAF among populations, with the largest MAF among Africans and Latinos (Figure S8). *CDH12* has been associated with angiogenesis and progression of several types of cancers.^52^, ^53^, ^54^ Specifically, in colorectal cancer, it has been suggested that *CDH12* increases cancer cell migration by promoting epithelial‐mesenchymal transition via activation of the Snail transcription factor pathway. *CDH12* expression is positively modulated by the chemotactic factor *CCL2*,^53^, ^54^ whose levels increases in blood and airway smooth muscle from asthma patients compared to healthy controls.^55^

We also attempted to assess previously associated loci for asthma exacerbations or moderate‐to‐severe asthma for association with asthma exacerbations in multiple ethnic groups. Although several variants showed association at *p* < .05, none surpassed the stringent Bonferroni correction, which could be due to differences in study design, phenotype definition, ethnicity, and clinical characteristics, among others. Of note, none of the previous findings was initially described in Asian or African populations, which highlights the need to increase ethnic diversity in genomic studies of asthma exacerbations.

Our study has several limitations. First, the *VCAM1*/*EXTL2* and *PANK1* loci did not surpass a stringent Bonferroni threshold of 4.7 × 10^−4^ (*p* = .05/106 variants) in the replication stage nor the genome‐wide significance in the combined analysis from all studies. Second, these loci exhibited modest effects sizes, which could impact the clinical relevance of these loci. Third, the history of asthma exacerbations was based on retrospective questionnaires in all cohorts but COMPASS, a randomized, prospective clinical trial. Fourth, to bring together large sample sizes necessary to map susceptibility variants, we considered studies where asthma exacerbations were reported for the previous 6 to 24 months or ever, which may have introduced some heterogeneity in the phenotype. Moreover, the replication stage comprised mostly European individuals, which hindered our capability to replicate associations driven in the discovery stage by non‐Europeans. Despite these limitations, our findings exhibited consistent effects for the *VCAM1*/*EXTL2* and *PANK1* loci independent of the time period assessed. Future studies in adequately powered and phenotypically harmonized cohorts should untangle the role of these loci in the time‐to‐first exacerbation, the annual number of exacerbations, or the temporal distance among events, explore other epigenetic mechanisms known to be involved in asthma (e.g., histone modifications or miRNAs),^56^ and the biological function of these genes. Moreover, although asthma exacerbation risk is influenced by sex in an age‐dependent manner,^57^ and our analyses were corrected for sex, future genome‐wide gene‐by‐sex interaction scans may reveal the influence of sex on the genetic susceptibility to exacerbations. On the other hand, we acknowledge several study strengths. First, we leveraged clinical and genetic data from 46,947 asthma patients from different ethnicities from 18 independent studies. Our study had statistical power ≥80% to detect associations with MAF > 17% and relative risk (RR) >1.20 in the discovery stage and for variants with MAF ≥ 1%, and was powered at 80% to detect associations with larger effect sizes (RR ≥ 1.85). Second, we identified novel, biologically plausible genetic factors of asthma exacerbations demonstrated by transcriptomics and epigenomics studies and evidence for prior literature. Moreover, we accounted for blood cell‐type heterogeneity to overcome the limitations of analyzing mixed cell types tissues.^56^, ^58^ Third, we evaluated previous genetic signals from asthma exacerbations in populations from several ancestries.

We identified suggestive loci for asthma exacerbations with consistent genetic effects across individuals from varying ancestral backgrounds using a multi‐ancestry approach. We also demonstrated that these loci are biologically functional and regulate RNA expression and adjacent CpG site DNA methylation as meQTL in whole blood cells. Our findings highlight *VCAM1*, *EXTL2*, and *PANK1* as functional loci for asthma exacerbations applicable to people across different ancestral backgrounds, warranting future investigation of these novel genomic mechanisms underlying asthma exacerbations.

## AUTHOR CONTRIBUTIONS

**Esther Herrera‐Luis:** Conceptualization (supporting); data curation (equal); formal analysis (lead); methodology (equal); writing original draft (lead); writing—review and editing (equal). **Victor E. Ortega:** Resources (equal); writing—review and editing (supporting); funding acquisition (lead). **Elizabeth J. Ampleford:** Resources (equal); data curation (equal); formal analysis (supporting); writing—review and editing (supporting). **Yang Yie Sio:** Resources (equal); investigation (supporting); data curation (equal); formal analysis (supporting); writing—review and editing (supporting). **Raquel Granell:** Resources (equal); data curation (equal); formal analysis (supporting); writing—review and editing (supporting). **Emmely de Roos:** Data curation (equal); formal analysis (supporting); writing—review and editing (supporting). **Natalie Terzikhan:** Data curation (equal); formal analysis (supporting); writing—review and editing (supporting). **Ernesto Elorduy Vergara:** Resources (equal); investigation (supporting); data curation (equal); formal analysis (supporting); writing—review and editing (supporting). **Natalia Hernandez‐Pacheco:** Investigation (supporting); data curation (equal); formal analysis (supporting); writing—review and editing (supporting). **Javier Perez‐Garcia:** Investigation (supporting); formal analysis (supporting); writing—review and editing (equal). **Elena Martin‐Gonzalez:** Investigation (supporting); writing—review and editing (supporting). **Fabian Lorenzo‐Diaz:** Resources (equal); conceptualization (supporting); funding acquisition (supporting). **Simone Hashimoto:** Resources (equal), data curation (equal); writing—review and editing (supporting). **Paul Brinkman:** Resources (equal); data curation (equal); writing—review and editing (supporting). **Andrea L. Jorgensen:** Data curation (equal); formal analysis (supporting); writing—review and editing (supporting). **Qi Yan:** Formal analysis (supporting). **Erick Forno:** Data curation (equal); formal analysis (supporting); writing—review and editing (supporting). **Susanne J. Vijverberg:** Resources (equal); data curation (equal); writing—review and editing (supporting). **Ryan Lethem:** Data curation (equal), writing—review and editing (supporting). **Antonio Espuela‐Ortiz:** Formal analysis (supporting), writing—review and editing (supporting). **Mario Gorenjak:** Resources (equal); investigation (supporting); data curation (equal); formal analysis (supporting); writing—review and editing (supporting). **Celeste Eng:** Investigation (supporting); writing—review and editing (supporting). **Ruperto González‐Pérez:** Resources (equal); investigation (supporting); writing—review and editing (supporting). **José M. Hernández‐Pérez:** Resources (equal); investigation (supporting); writing—review and editing (supporting). **Paloma Poza‐Guedes:** Resources (equal); investigation (supporting); writing—review and editing (supporting). **Olaia Sardón:** Resources (equal); investigation (supporting); writing—review and editing (supporting). **Paula Corcuera:** Resources (equal); investigation (supporting); writing—review and editing (supporting). **Greg A. Hawkins:** Investigation (supporting); writing—review and editing (supporting). **Annalisa Marsico:** Data curation (supporting); writing—review and editing (supporting). **Thomas Bahmer:** Investigation (supporting); writing—review and editing (supporting). **Klaus F. Rabe:** Investigation (supporting); writing—review and editing (supporting). **Gesine Hansen:** Investigation (supporting); writing—review and editing (supporting). **Matthias Volkmar Kopp:** Investigation (supporting); writing—review and editing (supporting). **Raimon Rios:** Formal analysis (supporting); writing—review and editing (supporting). **Maria Jesus Cruz:** Investigation (supporting); writing—review and editing (supporting). **Francisco‐Javier González‐Barcala:** Investigation (supporting); writing—review and editing (supporting). **José María Olaguibel:** Investigation (supporting); writing—review and editing (supporting). **Vicente Plaza:** Investigation (supporting); writing—review and editing (supporting). **Santiago Quirce:** Investigation (supporting); writing—review and editing (supporting). **Glorisa Canino:** Investigation (supporting); writing—review and editing (supporting). **Michelle Cloutier:** Investigation (supporting); writing—review and editing (supporting). **Victoria del Pozo:** Resources (supporting); investigation (supporting); writing—review and editing (supporting). **Jose R. Rodriguez‐Santana:** Investigation (supporting); writing—review and editing (supporting). **Javier Korta‐Murua:** Resources (equal); investigation (supporting); **Jesús Villar:** Conceptualization (supporting); resources (supporting); writing—review and editing (equal). **Uroš Potočnik:** Resources (equal); writing—review and editing (supporting). **Camila Figueiredo:** Resources (equal); data curation (equal); formal analysis (supporting); writing—review and editing (supporting). **Michael Kabesch:** Resources (equal); data curation (equal); writing—review and editing (supporting). **Somnath Mukhopadhyay:** Resources (equal); data curation (equal); writing—review and editing (supporting). **Munir Pirmohamed:** Resources (equal); data curation (equal); writing—review and editing (supporting). **Daniel B. Hawcutt:** Resources (equal); data curation (equal), writing—review and editing (supporting). **Erik Melén**: Resources (equal), writing—review and editing (supporting). **Colin N. Palmer:** Resources (equal), writing—review and editing (supporting). **Steve Turner:** Resources (equal), writing—review and editing (supporting). **Anke H. Maitland:‐van der Zee**: Resources (supporting), writing—review and editing (supporting). **Erika von Mutius:** Resources (equal); writing—review and editing (supporting). **Juan C. Celedón:** Resources (equal); writing—review and editing (supporting). **Guy Brusselle:** Resources (equal); writing—review and editing (supporting). **Fook Tim Chew:** Resources (equal); writing—review and editing (supporting). **Eugene Bleecker:** Resources (equal); writing—review and editing (supporting). **Deborah Meyers:** Resources (equal); writing—review and editing (supporting). **Esteban G. Burchard:** Resources (equal); funding acquisition (lead); supervision (supporting); writing—review and editing (supporting). **Maria Pino‐Yanes:** Resources (equal); conceptualization (lead); supervision (lead); funding acquisition (lead); methodology (equal); writing original draft (supporting); writing—review and editing (lead).

## CONFLICT OF INTEREST

AE‐O received grants from the Spanish Ministry of Science, Innovation, and Universities (MICIU) and Universidad de La Laguna (ULL). EH‐L, and MP‐Y report funding from the Spanish Ministry of Science and Innovation (MCIN/AEI/10.13039/501100011033) and by the European Social Fund “ESF Investing in your future” by the European Union. JP‐G reports funding from the Spanish Ministry of Universities. MP‐Y and FLD report grants from MCIN/AEI/10.13039/501100011033 and the European Regional Development Fund “ERDF A way of making Europe” by the European Union. MP‐Y reports grant support from GlaxoSmithKline, Spain paid to Fundación Canaria Instituto de Investigación Sanitaria de Canarias (FIISC) for a project outside the submitted work. MP‐Y and JV reports grants from Instituto de Salud Carlos III, Madrid, Spain. JV also reports funding by ISCIII and the European Regional Development Fund “ERDF A way of making Europe”. JMH‐P has received fees from CSL Behring, GSK, Astra‐Zeneca, laboratorios Menarini, Boehringer Ingelheim, FAES, laboratorios Esteve, Laboratorios Ferrer, Mundipharma, Laboratorios Rovi, Roche, Novartis, GRIFOLS, Pfizer, Acthelion‐Jansen, Chiesi y Laboratorios Bial for the realization of courses, talks, consultancies, and other activities related to his professional activity. FTC has received research support from the Singapore Ministry of Education Academic Research Fund, Singapore Immunology Network (SIgN), National Medical Research Council (NMRC) (Singapore), Biomedical Research Council (BMRC) (Singapore), and the Agency for Science Technology and Research (A*STAR) (Singapore). FTC has received consulting fees from Sime Darby Technology Centre; First Resources Ltd; Genting Plantation, and Olam International, outside the submitted work. YYS has received research support from the NUS Resilience & Growth Postdoctoral Fellowships. UP and MG received grants from the Ministry of Education, Science and Sport from Slovenia, the Slovenian Research Agency. M‐JC received grants from the Instituto de Salud Carlos III. DH received grant support from by NIHR for work on NIHR Alder Hey Clinical Research Facility, received payment for medicolegal report writing not related to asthma or pharmacogenomics for UK family court as an expert in pediatric clinical pharmacology. FJ‐B received fees from ALK, Astra‐Zeneca (AZ), Bial, Chiesi, Gebro Pharma, GlaxoSmithKline (GSK), Menarini, Rovi, Roxall, Sanofi, Stallergenes‐Greer and Teva. G‐B received fees from AZ, GSK, Boehringer‐Ingelheim, Novartis, Chiesi and Sanofi. JC received research materials from Pharmavite and GSK and Merck in order to provide medications free of cost to participants in NIH‐funded studies, unrelated to the current work. VO received grants from the National Heart, Lung, and Blood Institute, has participated in Data Safety Monitoring Boards for Regeneron and Sanofi, and participated as a Chair of the section on Genetics and Genomics of the American Thoracic Society. MVK has received grants from the German Federal Ministry of Education and Research, fees from Allergopharma GmbH, Sanofi Aventis GmbH, Infectopharm GmbH, Vertex GmbH, and Leti GmbH, has participated in Data Safety Monitoring Boards for Sanofi Aventis GmbH, and is the president of the German‐Swiss‐Austrian Society of Pediatric Pulmonology (GPP). NHP received support from the Instituto de Salud Carlos III, the European Social Funds from the European Union “ESF invests in your future,” the European Academy of Allergy and Clinical Immunology, and the European Respiratory Society. MP has received grants from NHS Chair of Pharmacogenetic grant from UK Department of Health, has received partnership funding for the following: MRC Clinical Pharmacology Training Scheme (co‐funded by MRC and Roche, UCB, Eli Lilly and Novartis); Joint PhD funding from EPSRC and AZ, and grant funding from Vistagen Therapeutics. He has also unrestricted educational grant support for the UK Pharmacogenetics and Stratified Medicine Network from Bristol‐Myers Squibb and UCB. He has developed an HLA genotyping panel with MC Diagnostics, but does not benefit financially from this. MP is part of the IMI Consortium ARDAT (www.ardat.org). SQ has received fees from GSK, AZ, Sanofi, Teva, Novartis, and Chiesi. SJ‐HV has received grants from SysPharmPediA EraNet. VdP has received fees from AZ and GSK. VP has received fees from Sanofi, AZ, Chiesi, MSD, and Boehringer Ingelheim, grant support from MSD, Chiesi Institutional, and Menarini. EvM has received grants from the German Federal Ministry of Education and Research and the Bavarian State Ministry of Health and Care, royalties/licenses from Elsevier GmbH, Georg Thieme Verlag, Springer‐Verlag GmbH and Elsevier Ltd. EvM has recieved fees from the Chinese University of Hongkong, European Commission, HiPP GmbH & Co KG, AZ, Imperial College London, Massachusetts Medical Society, Springer‐Verlag GmbH, Elsevier Ltd., Böhringer Ingelheim International GmbH, European Respiratory Society (ERS), Universiteit Utrecht, Faculteit Diergeneeskunde, Universität Salzburg, Springer Medizin Verlag GmbH, Japanese Society of Pediatric Allergy and Clinical Immunology (JSPACI), Klinikum Rechts der Isar, University of Colorado, Paul‐Martini‐Stiftung, Astra Zeneca, Imperial College London, Children´s Hospital Research Institute of Manitoba, Kompetenzzentrum für Ernährung (Kern), OM Pharma S.A., Swedish Pediatric Society for Allergy and Lung Medicine, Chinese College of Allergy and Asthma (CCAA), Verein zur Förderung der Pneumologie am Krankenhaus Großhansdorf e.V., Pneumologie Developpement, Mondial Congress & Events GmbH & Co. KG, American Academy of Allergy, Asthma & Immunology, Imperial College London, Margaux Orange, Volkswagen Stiftung, Böhringer Ingelheim International GmbH, European Respiratory Society (ERS), Universiteit Utrecht, Faculteit Diergeneeskunde, Österreichische Gesellschaft f. Allergologie u. Immunologie, Massachusetts Medical Society, OM Pharma S. A., Hanson Wade Ltd., iKOMM GmbH, DSI Dansk Borneastma Center, American Thoracic Society, HiPP GmbH & Co KG, Universiteit Utrecht, Faculteit Bètawetenschappen. EvM has patents No. PCT/EP2019/085016, EP21189353.2. 2021. and PCT/US2021/016918. 2021. pending, royalties paid to ProtectImmun for patent EP2361632 and patents EP1411977, EP1637147, and EP 1964570 licensed to ProtectImmun. EvM is a member of the EXPANSE Scientific Advisory Board, Member of the BEAMS External Scientific Advisory Board (ESAB), Member of the Editorial Board of “The Journal of Allergy and Clinical Immunology: In Practice”, Member of the Scientific Advisory Board of the Children's Respiratory and Environmental Workgroup (CREW), Member of the International Scientific & Societal Advisory Board (ISSAB) of Utrecht Life Sciences (ULS), University of Utrecht, Member of External Review Panel of the Faculty of Veterinary Science, University of Utrecht, Member of the Selection Committee for the Gottfried Wilhelm Leibniz Programme (DFG), Member of the International Advisory Board of Asthma UK Centre for Applied Research (AUKCAR), Member of the International Advisory Board of “The Lancet Respiratory Medicine”, Member of the Scientific Advisory Board of the CHILD (Canadian Healthy Infant Longitudinal Development) study, McMaster University, Hamilton, Canada, Asthma UK Centre for Applied Research and the Pediatric Scientific Advisory Board Iceland. The other authors declare no conflict of interest.

### PEER REVIEW

The peer review history for this article is available at https://publons.com/publon/10.1111/pai.13802.

## Supporting information

Table S1‐16

Appendix S1

## Data Availability

All data necessary to evaluate the conclusions of this manuscript are reported in the main text and/or the Appendix S1. Genome‐wide genotyping data for GALA II and SAGE are available at the database of Genotypes and Phenotypes (dbGaP) (Study Accession phs001274.v2.p1 and phs000092.v1.p1, respectively). The summary statistics of the multi‐ancestry discovery phase are available at the Zenodo repository: 10.5281/zenodo.5513443.
