# Supplementary material for: Multi‐ancestry genome‐wide association study of asthma exacerbations
Source: Pediatr Allergy Immunol. 2022 Jun 8;33(6):e13802. doi: 10.1111/pai.13802 (PMC9671132; doi:10.1111/pai.13802)
Supplement: Supplementary file 2 — Appendix S1 [file PAI-33-0-s002.docx]

**SUPPORTING INFORMATION**

**Multi-ancestry genome-wide association study of asthma exacerbations**

Esther Herrera-Luis^1^, Victor E. Ortega^2^, Elizabeth J. Ampleford^3^, Yang Yie Sio^4^, Raquel Granell^5^, Emmely de Roos^6,7^, Natalie Terzikhan^6,7^ , Ernesto Elorduy Vergara^8^, Natalia Hernandez-Pacheco^9,10^, Javier Perez-Garcia^1^, Elena Martin-Gonzalez^1^, Fabian Lorenzo-Diaz^1,11^, Simone Hashimoto^12^, Paul Brinkman^12^, U-BIOPRED Study Group^#^, Andrea l. Jorgensen^13^, Qi Yan^14^, Erick Forno^15^, Susanne J. Vijverberg^16,17,18^, Ryan Lethem^5^, Antonio Espuela-Ortiz^1^, Mario Gorenjak^19^, Celeste Eng^20^, Ruperto González-Pérez^21,22^, José M Hernández-Pérez^23,24^, Paloma Poza-Guedes^21,22^, Olaia Sardón^25,26^, Paula Corcuera^25^, Greg A. Hawkins^27^, Annalisa Marsico^28^, Thomas Bahmer^29^, Klaus F. Rabe^29^, Gesine Hansen^30^, Matthias Volkmar Kopp^31,32,33^, Raimon Rios^34^, Maria Jesus Cruz^10,35^, Francisco-Javier González-Barcala^36^, José María Olaguibel^10,37^, Vicente Plaza^10,38^, Santiago Quirce^10,39^, Glorisa Canino^40^, Michelle Cloutier^41^, Victoria del Pozo^10,42^, Jose R Rodriguez-Santana^43^, Javier Korta-Murua^26^, Jesús Villar^10,44^, Uroš Potočnik^45^, Camila Figueiredo^46^, Michael Kabesch^47^, Somnath Mukhopadhyay^48,49^, Munir Pirmohamed^50^, Daniel B. Hawcutt^51,52,53^, Erik Melén^9,54^, Colin N Palmer^49^, Steve Turner^55^, Anke H. Maitland-van der Zee^16,17,18^, Erika von Mutius^56,57,58^, Juan C. Celedón^15^, Guy Brusselle^6,7,59^, Fook Tim Chew^4^, Eugene Bleecker^60^, Deborah Meyers^60^, Esteban G Burchard^22,61^, Maria Pino-Yanes^1,10,62^

From:

*^1^Genomics and Health Group, Department of Biochemistry, Microbiology, Cell Biology and Genetics, Universidad de La Laguna (ULL), San Cristóbal de La Laguna, Tenerife, Spain;*

*^2^Department of Internal Medicine, Division of Respiratory Medicine, Mayo Clinic, Scottsdale, Arizona, U.S.A;*

*^3^Department of Internal Medicine, Center for Precision Medicine, Wake Forest School of Medicine, Winston-Salem, North Carolina, U.S.A.;*

*^4^Department of Biological Sciences, National University of Singapore, Singapore;*

*^5^MRC Integrative Epidemiology Unit (IEU), Population Health Sciences, Bristol Medical School, University of Bristol, Oakfield Grove, Bristol, U.K.;*

*^6^Department of Epidemiology, Erasmus University Medical Center, Rotterdam, The Netherlands;*

*^7^Department of Respiratory Medicine, Ghent University Hospital, Ghent, Belgium;*

*^8^Institute of Computation Biology, Helmholtz Zentrum München, German Research Center for Environmental Health, Munich, Germany;*

*^9^Department of Clinical Sciences and Education, Södersjukhuset, Karolinska Institutet, Stockholm, Sweden;*

*^10^CIBER de Enfermedades Respiratorias (CIBERES), Madrid, Spain;*

*^11^Instituto Universitario de Enfermedades Tropicales y Salud Pública de Canarias (IUETSPC), Universidad de La Laguna (ULL), San Cristóbal de La Laguna, Tenerife, Spain;*

*^12^Dept. Respiratory Medicine, Amsterdam University Medical Center, University of Amsterdam, Amsterdam, the Netherlands;*

*^13^14Department of Health Data Science, Institute of Population Health, University of Liverpool, L69 3GL, Liverpool, U.K.;*

*^14^Department of Obstetrics and Gynecology, Columbia University Irving Medical Center, New York, New York, U.S.A.;*

*^15^Division of Pediatric Pulmonary Medicine, UPMC Children's Hospital of Pittsburgh, University of Pittsburgh, Pittsburgh, Pennsylvania, U.S.A.;*

*^16^Dept of Respiratory Medicine, Amsterdam UMC, University of Amsterdam, Amsterdam, The Netherlands;*

*^17^Division of Pharmacoepidemiology and Clinical Pharmacology, Faculty of Science, Utrecht University, Utrecht, The Netherlands;*

*^18^Dept of Paediatric Respiratory Medicine and Allergy, Emma's Children Hospital, Amsterdam UMC, University of Amsterdam, Amsterdam, The Netherlands;*

*^10^Center for Human Molecular Genetics and Pharmacogenomics, Faculty of Medicine, University of Maribor, Maribor, Slovenia.*

*^20^Department of Medicine, University of California San Francisco, San Francisco, California, U.S.A.;*

*^21^Allergy Department, Hospital Universitario de Canarias, Santa Cruz de Tenerife, Tenerife, Spain;*

*^22^Severe Asthma Unit, Allergy Department, Hospital Universitario de Canarias, Santa Cruz de Tenerife, Tenerife, Spain*

*^23^Pulmonary Medicine, Hospital Universitario de N.S de Candelaria, Santa Cruz de Tenerife, Spain;*

*^24^Pulmonary Medicine, Hospital General de La Palma, La Palma, Santa Cruz de Tenerife, Spain;*

*^25^Division of Pediatric Respiratory Medicine, Hospital Universitario Donostia, San Sebastián, Spain; ^26^Department of Pediatrics, University of the Basque Country (UPV/EHU), San Sebastián, Spain;*

*^27^Department of Biochemistry, Wake Forest School of Medicine, Winston-Salem, North Carolina, U.S.A.;*

*^28^Computational Health Center, Helmholtz Zentrum München, German Research Center for Environmental Health, Munich, Germany;*

*^29^LungenClinic Grosshansdorf, Grosshansdorf and Dept of Medicine, Christian Albrechts University Kiel, Germany; Members of the German Center for Lung Research (DZL)*

*^30^Department of Pediatric Pneumology, Allergology and Neonatology, Hannover Medical School, Hannover, Germany; Members of the German Center for Lung Research (DZL);*

*^31^Division of Pediatric Pneumology & Allergology, University Medical Center Schleswig-Holstein, Lübeck, Germany;*

*^32^Airway Research Center North (ARCN), Members of the German Center for Lung Research (DZL);*

*^33^Department of Paediatric Respiratory Medicine, Inselspital, University Children's Hospital of Bern, University of Bern, Bern, Switzerland;*

*^34^Programa de Pós Graduação em Imunologia (PPGIm), Instituto de Ciências da Saúde, Universidade Federal da Bahia (UFBA), Salvador, Brazil;*

*^35^Servicio de Neumología, Hospital Vall d’Hebron, Barcelona, Spain;*

*^36^Servicio de Neumología, Complejo Hospitalario Universitario de Santiago, Santiago de Compostela, Spain;*

*^37^Servicio de Alergología, Complejo Hospitalario de Navarra, Pamplona, Navarra, Spain;*

*^38^Departamento de Medicina Respiratoria, Hospital de la Santa Creu i Sant Pau, Instituto de Investigación Biomédica Sant Pau (IIB Sant Pau), Barcelona, Spain;*

*^39^Department of Allergy, La Paz University Hospital, IdiPAZ, Madrid, Spain;*

*^40^Behavioral Sciences Research Institute, University of Puerto Rico, San Juan, Puerto Rico;*

*^41^Department of Pediatrics, University of Connecticut, Farmington, Connecticut, U.S.A;*

*^42^Immunology Department, Instituto de Investigación Sanitaria Hospital Universitario Fundación Jiménez Díaz, Madrid, Spain;*

*^43^Centro de Neumología Pediátrica, San Juan, Puerto Rico;*

*^44^Multidisciplinary Organ Dysfunction Evaluation Research Network, Research Unit, Hospital Universitario Dr. Negrín, Las Palmas de Gran Canaria, Spain*

*^45^Laboratory for Biochemistry, Molecular Biology and Genomics, Faculty for Chemistry and Chemical Engineering, University of Maribor, Maribor, Slovenia;*

*^46^Instituto de Ciências da Saúde, Universidade Federal da Bahia, Salvador, Brazil;*

*^47^Dept of Paediatric Pneumology and Allergy, University Children's Hospital Regensburg (KUNO), Regensburg, Germany;*

*^48^Academic Dept of Paediatrics, Brighton and Sussex Medical School, Royal Alexandra Children's Hospital, Brighton, UK;*

*^49^Population Pharmacogenetics Group, Biomedical Research Institute, Ninewells Hospital and Medical School, University of Dundee, Dundee, UK;*

*^50^Dept of Pharmacology and Therapeutics, Institute of Systems, Molecular and Integrative Biology, University of Liverpool, Liverpool, UK;*

*^51^Dept of Women's and Children's Health, University of Liverpool, Liverpool, UK;*

*^52^Alder Hey Children's Hospital, Liverpool, UK;*

*^53^NIHR Alder Hey Clinical Research Facility, Alder Hey Children's Hospital, Liverpool, UK;*

*^54^Sachs’ Children’s Hospital, South General Hospital, Stockholm, Sweden;*

*^55^Child Health, University of Aberdeen, Aberdeen, UK;*

*^56^Institute for Asthma and Allergy Prevention, Helmholtz Zentrum München, German Research Center for Environmental Health, Munich, Germany;*

*^57^Dr von Hauner Children's Hospital, LMU München, Munich, Germany;*

*^58^Comprehensive Pneumology Center Munich (CPC-M), Member of the German Center for Lung Research, Munich, Germany;*

*^59^Department of Respiratory Medicine, Erasmus University Medical Center, Rotterdam, The Netherlands;;*

*^60^Department of Internal Medicine, Division of Genetics, Genomics, and Precision Medicine, University of Arizona College of Medicine, Tucson, Arizona, U.S.A.;*

*^61^Department of Bioengineering and Therapeutic Sciences, University of California San Francisco, San Francisco, California, U.S.A.;*

*^62^Instituto de Tecnologías Biomédicas (ITB), Universidad de La Laguna (ULL), San Cristóbal de La Laguna, Tenerife, Spain.*

*#The members of the U-BIOPRED Study Group are provided in the online data supplement.*

***Corresponding author:**

Dr. Maria Pino-Yanes

Genomics and Health Group, Department of Biochemistry, Microbiology, Cell Biology and Genetics. Universidad de La Laguna (ULL)

Apartado 456, 38200, San Cristóbal de La Laguna, Tenerife, Spain

Phone: (+34) 922 316502 6343

Fax: (+34) 922 318 490

e-mail: mdelpino@ull.edu.es

**SUPPORTING INFORMATION**

**Studies included in the analysis**

Asthma patients from different asthma cohorts, case-control studies of asthma, clinical trials, and population-based studies were analysed. Most childhood studies were part of the Pharmacogenomics in Childhood Asthma (PiCA) consortium (1). Asthma exacerbations were defined as a binary categorical variable based on the presence of asthma-related hospitalizations, unexpected and/or unscheduled asthma care and/or oral corticosteroids use in the last 6 to 24 months or ever, depending on the data available for each study. In the replication stage, school absences were also considered for the definition of exacerbations. In the multi-ethnic discovery phase, we included predominantly European or European-descent, Singaporean Chinese, Hispanic/Latino, and African American patients with asthma. In the replication phase, predominantly European individuals, Hispanics/Latinos, and Filipinos with asthma were assessed. In the discovery stage, we included individual GWAS that met a minimum threshold of 50 cases and 50 controls for statistical robustness, except for the East Asian group, where only one study of the aforementioned ethnic group was available to be included (**Table S1**). In the replication stage, we considered a less stringent threshold based on the availability of the data (**Table S2**). A total of 12 and 11 studies were included in the discovery and replication phases, respectively.

***Discovery phase***

ALSPAC

The Avon Longitudinal Study of Parents and Children (ALSPAC) is a birth cohort that recruited pregnant women in Avon (United Kingdom). Data from parents and children were regularly collected since the child was born during research clinic assessments. The main purpose of the follow-up phase of this cohort is to study the transition from childhood into adulthood of those children. This study includes a wide variety of phenotypic, environmental, genetic, and epigenetic information from children. Further details about the data available, recruitment criteria, and strategy are available elsewhere (2–4).

Pregnant women residents in Avon (United Kingdom) with expected dates of delivery 1^st^ April 1991 to 31^st^ December 1992 were invited to take part in the study. The initial number of pregnancies enrolled is 14,541 (for these at least one questionnaire has been returned or a “Children in Focus” clinic had been attended by 19/07/99). Of these initial pregnancies, there was a total of 14,676 foetuses, resulting in 14,062 live births and 13,988 children who were alive at 1 year of age.

When the oldest children were approximately 7 years of age, an attempt was made to bolster the initial sample with eligible cases who had failed to join the study originally. As a result, when considering variables collected from the age of seven onwards (and potentially abstracted from obstetric notes) there are data available for more than the 14,541 pregnancies mentioned above. The number of new pregnancies not in the initial sample (known as Phase I enrolment) that are currently represented on the built files and reflecting enrolment status at the age of 24 is 913 (456, 262 and 195 recruited during Phases II, III and IV respectively), resulting in an additional 913 children being enrolled. The phases of enrolment are described in more detail in the cohort profile paper and its update. The total sample size for analyses using any data collected after the age of seven is therefore 15,454 pregnancies, resulting in 15,589 foetuses. Of these 14,901 were alive at 1 year of age.

A 10% sample of the ALSPAC cohort, known as the Children in Focus (CiF) group, attended clinics at the University of Bristol at various time intervals between 4 to 61 months of age. The CiF group was chosen at random from the last 6 months of ALSPAC births (1432 families attended at least one clinic). Excluded were those mothers who had moved out of the area or were lost to follow-up, and those partaking in another study of infant development in Avon.

Ethical approval for the study was obtained from the ALSPAC Ethics and Law Committee and the Local Research Ethics Committees. Further details are available in the cohort profile articles and the study website contains details of all the data that is available through a fully searchable data dictionary and variable search tool: http://www.bristol.ac.uk/alspac/researchers/our-data/. Informed consent for the use of data collected via questionnaires and clinics was obtained from participants following the recommendations of the ALSPAC Ethics and Law Committee at the time.

The ALSPAC children were genotyped on the Illumina HumanHap550-Quad platform, by the Wellcome Trust Sanger Institute, Cambridge (United Kingdom) and the Laboratory Corporation of America, Burlington, NC, using support from 23andMe.

The UK Medical Research Council and Wellcome (Grant ref: 102215/2/13/2) and the University of Bristol provide core support for ALSPAC. This publication is the work of the authors and Raquel Granell will serve as a guarantor for the contents of this paper.

Individuals with doctor ever diagnosed asthma at 18 years and either ‘hospital admission for asthma or wheeze between 12 and 14 years’ or ‘at least 1 school absence for asthma in past 12 months at 14 years’ were considered as cases. Individuals with doctor ever diagnosed asthma at 18 years and negative answers for the two previous questions were considered as controls.

BREATHE

The BREATHE study recruited children and young adults (aged 3-22 years old) with physician-diagnosed asthma at primary and secondary care units from the United Kingdom (5–7). Quality control of genome-wide genotyping data obtained with the Axiom Precision Medicine Research Array has been described elsewhere (8). BREATHE was approved by the Tayside Committee on Medical Research Ethics (Dundee, United Kingdom). Cases were defined as patients with asthma and asthma-related hospitalizations and/or emergency room visits in the last 6 months, whereas controls comprised asthma patients that did not report asthma-related hospitalizations or emergency room visits.

COMPASS

COMPASS is a 6-month randomized double-blind clinical trial with adults and adolescents with a diagnosis of asthma on budesonide/formoterol for maintenance. Patients using systemic corticosteroids or respiratory infections 30 days prior to enrolment were excluded. Patients monitored their symptoms and use of medication daily. Spirometry, the Asthma Control Questionnaire, and Asthma Quality of Life Questionnaire were evaluated at clinic visits (9).

Treatment stepwise management in COMPASS included three steps: A) Budesonide/formoterol 160/4.5 µg/inhalation Turboinhaler (TBH) *bis in die* (bid) + placebo to Seretide pressurized metered-dose inhaler (pMDI) two inhalations bid and as needed budesonide/formoterol 160/4.5 µg/inhalation TBH inhaled corticosteroids (ICS) and long-acting bronchodilator inhalers (LABA) (turboinhaler); B) Budesonide/formoterol 320/9 µg/inhalation TBH bid + placebo to Seretide pMDI two inhalations bid and as needed Terbutaline Sulphate 0.4 mg/inhalation TBH; C) Seretide pMDI 25/125 µg/inhalation two inhalations bid and placebo to Budesonide/formoterol TBH bid and as needed Terbutaline Sulphate 0.4 mg/inhalation TBH.

Individuals of European descent genotyped with the Illumina HumanOmniExpress-12v1_C were considered in the discovery stage.

GALA II

The Genes-Environment and Admixture in Latino Americans (GALA II) study is a cross-sectional case-control study of asthma. All subjects (aged 8-22 years) must have four Latino grandparents. Participants were recruited from five different centres in the United States (Chicago, Illinois; New York City, New York; Houston, Texas; San Francisco, California) and Puerto Rico (San Juan) (10–12). GALA II was approved (ethics approval number: 217802) by The Human Research Protection Program Institutional Review Board of the University of California, San Francisco (San Francisco, United States). Genome-wide genotyping and quality control is detailed elsewhere (13). Cases were defined as patients with asthma and asthma-related hospitalizations, acute asthma care visits and/or use of oral corticosteroids in the previous 12 months, whereas controls comprised asthma patients that did not report asthma-related hospitalizations, acute asthma care visits or use of oral corticosteroids for the same period of time.

GoSHARE

Genetic of Scottish Health Research Register (GoSHARE) study recruited children and young adults (aged 3 to 18 years old) from Tayside (Scotland) who consented to blood donation for medical research. Patients were identified from the National Health Service databases (14). Methods for quality control of genome-wide genotyping data are detailed elsewhere (8). GoSHARE was approved by the Tayside Committee on Medical Research Ethics (Dundee, United Kingdom). Cases comprised patients with asthma and asthma-related hospitalizations, emergency room visits, and/or use of oral corticosteroids in the previous 12 months whereas controls included asthma patients that did not report asthma-related hospitalizations, emergency room visits, or use of oral corticosteroids for the same period of time.

PACMAN

The Pharmacogenetics of Asthma Medication in Children: Medication with Anti-inflammatory effects (PACMAN) study is a cross-sectional retrospective study of children (aged 4-12 years) that reported use of any asthma medication through the pharmacy records in the Netherlands. Additional health information was requested through the general practitioner and by questionnaire (15). PACMAN was approved (protocol number: 08/023) by The Medical Ethics Committee of the University Medical Centre Utrecht (Utrecht, the Netherlands). Methods for quality control of genome-wide genotyping data are detailed elsewhere (16). Cases were defined as patients with asthma and asthma-related emergency room visits and/or use of oral corticosteroids in the previous 12 months. Controls comprised asthma patients that did not report any of the events mentioned above for the same period of time.

PAGES

The Paediatric Asthma Gene-Environment Study (PAGES) is a cross-sectional study of children and adolescents (aged 2-16 years old) with paediatrician-diagnosed asthma recruited in hospitals in Scotland between 2008 and 2011. Participants underwent clinical assessment, consented to the donation of saliva samples, and filled dietary and quality of life questionnaires (17). PAGES was approved by the Cornwall and Plymouth Research Ethics Committee (Plymouth, United Kingdom). Genome-wide genotyping data and quality control have been described elsewhere (8). Cases were defined as patients with asthma and asthma-related hospitalizations, and/or use of oral corticosteroids in the previous 6 months, whereas controls comprised asthma patients that did not report asthma-related hospitalizations or use of oral corticosteroids for the same period of time.

PASS

Children and young adults (aged 5 to 18 years old) from several centres at the United Kingdom with asthma requiring corticosteroid therapy under paediatrician supervision were recruited as part of the Pharmacogenetics of Adrenal Suppression study (PASS). Inclusion criteria included a diagnosis of asthma and clinical concern about adrenal suppression (18,19). PASS was approved by the Liverpool Paediatric Research Ethics Committee (Liverpool, United Kingdom) (reference number: 08/H1002/56). Methods for genotyping and quality control are detailed elsewhere (19). Cases were defined as patients with asthma and use of oral corticosteroids in the previous 6 months, whereas controls comprised asthma patients that did not report oral corticosteroids use for the same period of time.

SLOVENIA

SLOVENIA is a case-control study of asthma in children and young adults (aged 5-18 years old). Asthma was defined by physician diagnosis and hospital records according to the American Thoracic Society (ATS) criteria. Patients with mild and moderate persistent asthma were recruited from tertiary health centres in Slovenia (20). This study was approved by the Slovenian National Medical Ethics Committee (Ljubljana, Slovenia). Genome-wide genotyping and quality control is detailed elsewhere(8). Cases were defined as patients with asthma and use of asthma-related hospitalizations, emergency room visits, and/or use of oral corticosteroids in the previous 6 months. Controls comprised asthma patients that did not report asthma-related hospitalizations, emergency room visits, or use of oral corticosteroids for the same period of time.

SAGE

The Study of African Americans, Asthma, Genes and & Environments (SAGE) is a cross-sectional case-control study of asthma that enrolled children and young adults aged 8 to 21 years with four grandparents of African American ancestry. Recruitment was conducted at the San Francisco Bay Area, California, United States (10–12). SAGE was approved (ethics approval number: 210362) by The Human Research Protection Program Institutional Review Board of the University of California, San Francisco (San Francisco, United States). Genome-wide genotyping and quality control is detailed elsewhere (13). Cases were defined as patients with asthma and asthma-related hospitalizations, acute asthma care visits, and/or use of oral corticosteroids in the previous 12 months whereas. Controls were asthma patients that did not report asthma-related hospitalizations, acute asthma care visits, or use of oral corticosteroids for the same period of time.

SCSGES

Singapore Cross Sectional Genetic Epidemiology Study (SCSGES) is an ongoing cross-sectional genetic epidemiology study on allergic diseases among Singapore Chinese individuals (age: 6-57 years) (21–25). Participants were of Chinese ethnicity and resident in Singapore, and their DNA was extracted from mouthwash and blood samples. Ethnicity was determined based on participant’s self-reported information and confirmed by principal component analysis in the previous study (21). Asthma case was defined by ever having symptoms positively diagnosed by a physician. Study approval was obtained from the Institutional Review Board of the National University of Singapore (NUS-IRB Ref-Code: 07–023, 09–256, 10–445, 13–075, B-10-343, and H-18-036) and the Institutional Review Board of the National Healthcare Group Domain, Specific Review Board (B/04/055). Methods for quality control of genotyping data are described elsewhere (22). Cases were defined as patients with asthma and asthma-related hospitalizations, and/or acute asthma care visits in the previous 12 months, whereas controls comprised asthma patients that did not report asthma-related hospitalizations or acute asthma care visits for the same period of time.

U-BIOPRED

The Unbiased Biomarkers for the Prediction of Respiratory Disease Outcomes (U-BIOPRED) is a prospective cohort study of participants with severe asthma that underwent supervision by a respiratory physician for at least 6 months. Recruitment was performed at clinical centres in Europe. Further details about the study and the quality control of genotyping data are described elsewhere (26,27). The study was approved by the ethics committee for each participating clinical institution. Cases were defined as patients with asthma and asthma-related hospitalizations, acute asthma care visits and/or use of oral corticosteroids in the previous 12 months, whereas controls comprised asthma patients that did not report asthma-related hospitalizations, acute asthma care visits or use of oral corticosteroids for the same period of time.

***Replication stage***

ALLIANCE

“The ALL Age Asthma Cohort (ALLIANCE) of the German Center for Lung Research (DZL) is a prospective, multi-centre, observational cohort study with seven recruiting sites across Germany. Data are derived from four sources: (a) patient history from medical records, (b) standardized questionnaires and structured interviews, (c) telephone interviews, and (d) objective measurements. Objective measurements include, amongst others, lung function and quantitative assessment of airway inflammation and exhaled breath, peripheral blood, skin, nasal, pharyngeal, and nasopharyngeal swabs, nasal secretions, primary nasal epithelial cells, and induced sputum. In cases, objective measurements and biomaterial collection are performed regularly, while control subjects are only examined once at baseline” (28). The ALLIANCE cohort has recruited more than 1,000 individuals, including asthma patients and healthy subjects with ages ranging from six months to 84 years. After baseline examination, a follow-up of patients with asthma is performed generally once a year (29). ALLIANCE was approved by the local ethics committees.

DNA from individuals was extracted from peripheral blood samples collected in EDTA tubes (S-Monovette 1.2 ml with K+EDTA) from Sarstedt (Nümbrecht, Germany). Genotyping was conducted using the Infinium Global Screening Array-24 v1.0 +MD BeadChip at the Genome Analysis Center (GAC) of Helmholtz Zentrum München. The raw plink files contained 712,189 variants and 911 samples. Genomic coordinates were consistent with the Genome Reference Consortium Human Build 37(GRCh37, hg19). Genotype data was pre-processed in order to remove single nucleotide polymorphisms (SNPs) not encoded as single nucleotides in alphabet ‘ACGT’ (651,582 variants remained), those with genotyping call rates lower than 97% (630,354 variants and 886 samples remained), those with minor allele frequency lower than 1% (480,262 variants remained) and those duplicated (479,972 SNPs remained). Regarding sample exclusion, the following criteria were considered: discordance between reported sex and genetic sex estimated based on chromosome X homozygosity rate (885 samples remained), outliers of heterozygosity rates (859 samples remained), duplicated individuals, and related individuals according to Pi-hat value >0.2 (830 samples remained). For duplicated individuals or SNPs, those with the least missingness were retained. For each pair of related individuals, the individual with the highest call rate was retained. Deviation of the Hardy-Weinberg equilibrium, based on threshold of *p*<1x10^-10^, was assessed using control samples (479,807 variants remained). Based on the first ten components from multidimensional scaling (MDS), a total of eight individuals were identified as genetic outliers. After quality control, a total of 822 remained for analysis. Cases comprised asthma patients that reported asthma-related emergency room visits, hospitalizations, and/or use of oral corticosteroids in the previous year. Controls were defined as patients with asthma but no report of the events mentioned above for the same period of time.

BAMSE

The Children Allergy Milieu Stockholm an Epidemiological Study (BAMSE) is a prospective population-based birth cohort in which newborn infants between 1994 and 1996 from the north and central area of Stockholm were recruited. The baseline questionnaires were obtained when the children were about 2 months old and follow-up have been conducted at 1, 2, 4, 8, 16, and 24 years old (30,31). BAMSE was approved by the Regional ethical committee in Stockholm (Stockholm, Sweden) (ethics approval numbers: 02-420 and 2010/1474-31/3). DNA was extracted from peripheral blood and genotyping is described elsewhere (32). Recently, a total of 2,378 16-year-old children were additionally genotyped with the Illumina Infinium Global Screening Array-24 .10 BeadChip following the same sample collection procedures. Quality control (QC) was performed following the Ricopili pipeline (33). Data was imputed using the Haplotype Reference Consortium 1.1 reference panel (34) by means of IMPUTE 2 (35). The results from the two genotyping waves were meta-analyzed together to be included in the replication stage. For the current study, clinical and demographic data was obtained from questionnaires at 8 years old. Cases comprised asthma patients that reported asthma-related emergency room visits, hospitalizations and/or school absences in the previous year whereas controls were defined as patients with asthma but no report of the events mentioned above for the same period of time.

BREATHE

Individuals from the BREATHE study with exacerbation data and genotyped with the Illumina Infinium CoreExome-24 BeadChip (Illumina) were included in the replication stage. Quality control has been described elsewhere (8). Individuals from BREATHE that had been included in the subset of individuals genotyped with the Axiom Precision Medicine Research Array and analysed in the discovery stage were removed from analysis in the replication stage. BREATHE was approved by the Tayside Committee on Medical Research Ethics (Dundee, United Kingdom). Cases comprised asthma patients that reported use of oral corticosteroids, asthma-related hospitalizations, and/or school absences in the last 6 months, whereas controls were defined as patients with asthma but no report of the events mentioned above for the same period of time.

COMPASS

To increase the ethnic diversity in the replication stage, individuals with asthma exacerbations data available from the COMPASS study that reported Filipino ethnicity and were recruited in Philippines (COMPASS PHI) were included in the analysis.

FollowMAGICS

As part of the Multicenter Asthma Genetics in Childhood Study (MAGICS), children with asthma were recruited at seven centres from Germany and Austria (Wesel, Bochum Cologne, Freiberg, Munich, Feldkirch, and Vienna). Asthma was diagnosed by a paediatric pulmonologist or allergologist based on the examination, history and tests of lung function. Children and young adults from 7 to 25 years with persistence of asthma symptoms were included in the follow-up phase of MAGICS (followMAGICS) (36–38). FollowMAGICS was approved (ethics reference number: 01218) by the Ethik-Kommission der Bayerischen Landesärztekammer (Munich, Germany). Cases were defined as patients with asthma and asthma-related hospitalizations, emergency room visits, and/or other unexpected and/or unscheduled acute asthma care visits in the previous 12 months, whereas controls comprised asthma patients that did not report any of the events mentioned above for the same period of time.

GEMAS

The Genomics and Metagenomics of Asthma Severity (GEMAS) study is a multicentre study that recruited asthma patients aged 8 to 82 years from the allergy and pulmonary medicine units from several hospitals in the Canary Islands and Donostia, Spain. The case/control status was defined based on the presence/absence of oral corticosteroid use, emergency room visits, and/or asthma-related hospitalizations in the year prior to enrolment. Demographic and clinical variables were collected using a standardized questionnaire (39). The study was approved by the ethics committees of participant centres (approval 29/17 for the Canary Islands hospitals and PI2019077 for Hospital Universitario Donostia).

DNA from individuals was extracted from peripheral blood samples using the Illustra blood genomicPrep Mini Spin Kit (GE Healthcare, Amersham, UK) and stored at -20ºC. Genotyping was conducted with the Global Screening Array v3.0 (Illumina) at the Genotyping National Centre (CeGEN) in Madrid (Spain). Allele calling was performed with the Infinium GSA v3.0 Manifest File (GRCh37; version of Aug 12, 2019) via GenomeStudio 2.0. Only non-zeroed SNPs (650,181 SNPs) were exported into PED/MAP files format. Genotype data were pre-processed to remove SNPs not assigned to autosomal and sexual chromosomes, duplicated, or monomorphic (543,596 SNPs remained). Moreover, genetic variants with a genotyping call rate lower than 95% were removed (445 variants). All batches from these studies were subjected to quality control together using R 4.0.2 (40) and PLINK v1.9 (41). Samples were discarded if they a) had a genotyping call rate lower than 95%; b) showed discordance between reported sex and genetic sex estimated based on chromosome X homozygosity rate estimates; or c) represented duplicated individuals or related individuals according to Pi-hat value >0.2. For each pair of related individuals, the individual with the highest call rate was retained. Genetic outliers were excluded according to principal component analysis. No variants were removed due to Hardy-Weinberg equilibrium in these studies with only asthma patients during the QC, but variants assessed for association were confirmed to be in Hardy-Weinberg equilibrium using mid p-values (42). From a total of 288 individuals from GEMAS genotyped, 282 remained for analysis.

Cases were defined as patients with asthma and asthma-related hospitalizations, emergency room visits, and/or use of oral corticosteroids in the previous 12 months, whereas controls comprised asthma patients that did not report any of the events mentioned above for the same period of time.

HPR

The Hartford-Puerto Rico (HPR) study is a case–control study of asthma in Puerto Rican children recruited in Hartford (Connecticut, United States) and San Juan (Puerto Rico). Participants were eligible if they had 6 to 14 years and four Puerto Rican grandparents. Asthma was defined as physician-diagnosed asthma and at least one episode of wheeze in the year prior enrolment (43). Details on the definition of the phenotype, quality control, and statistical analysis have been published elsewhere (44). Briefly, a total of 236 exacerbators and 318 non-exacerbators were included in the analysis. Asthma exacerbation status was defined as one or more asthma-related hospitalizations or emergency room visits in the previous year requiring systemic corticosteroids, or a course of systemic corticosteroids in the previous year. The variants that showed suggestive association in the discovery phase were evaluated in previously published GWAS results with adjustment for age, sex, inhaled steroid use, and the first two principal components (44).

MEGA

The Mechanism underlying the genesis and evolution of asthma (MEGA) project is a prospective multi-center cohort study of adults including asthma patients and healthy controls (aged 18-75 years) recruited from eight university hospitals in Spain. Individuals were included in the study if they had a diagnosis of asthma based on Global Initiative for Asthma (GINA) criteria for at least 1 year before enrollment. Individuals with a report of other acute or chronic active lung disorders, or significant psychiatric disorders were considered ineligible (45). This study was approved by the Clinical Research Ethics Committee of participating centers. Genotype data quality control was performed following the same procedures described for GEMAS. From a total of 168 individuals from MEGA genotyped, 163 individuals from MEGA were kept for further analyses. Cases were defined as patients with asthma and asthma-related hospitalizations, emergency room visits, and/or use of oral corticosteroids in the previous 12 months, whereas controls comprised asthma patients that did not report any of the events mentioned above for the same period of time.

The Rotterdam Study

The Rotterdam Study is a prospective, population-based cohort study including adults in Rotterdam (The Netherlands). The initial recruitment in 1990 included adults aged 45 years old or over (RSI). Three follow-up phases have been performed in 2000 (RSII), 2006 (RSIII), and 2016 (RSIV). RSIV recruited individuals aged 40 years old or over (46). The Rotterdam Study was approved by the Medical Ethics Committee of the Erasmus MC and by the Ministry of Health, Welfare, and Sport of the Netherlands, implementing the Wet Bevolkingsonderzoek: ERGO (Population Studies Act: Rotterdam Study). The results from the three follow-up phases were meta-analysed together to be included in the replication stage. Cases were defined as patients with asthma and asthma-related emergency room visits in the previous 12 months, whereas controls comprised asthma patients that did not report asthma-related emergency room visits for the same period of time. The current analysis comprised individuals aged 55.6-96.4 (RS1), 56.1-100.1 (RS2), 46.2-88.8 (RS3).

SCAALA

The Social Changes, Asthma and Allergy in Latin America (SCAALA) study is a longitudinal study of asthma and allergic diseases of Brazilian children (47). Children who reported asthma symptoms and had available data on emergency room visits or hospitalizations due to wheeze in the previous year were included in the analysis. Details on the definition of the phenotype, quality control, and statistical analysis have been published elsewhere (44). A total of 256 children with asthma aged 5-12 years, including 139 exacerbators, were examined in the replication phase. Asthma exacerbations were defined as one or more hospitalization or emergency room visits due to wheezing in the previous year. The variants that showed suggestive association in the discovery phase were evaluated in previously published GWAS results with adjustment for age, sex, and the first two principal components (44).

UK Biobank

The UKB is a prospective population-based cohort that recruited subjects from across the United Kingdom (48). Details on the definition of the phenotype, quality control, and statistical analysis have been published elsewhere (49). The doctor’s diagnosis of asthma (International Classification of Diseases 10 code J45 or J46) was extracted from the UKB data release from March 2019. Study participants aged 40- 70 years were recruited in 2006-2010, when most completed a baseline visit. Briefly, a total of 34,167 adults, including 1,658 exacerbators, were considered for analysis. Exacerbations were defined based on the presence of asthma and at least one asthma-related hospitalization, while non-exacerbators had asthma but no asthma hospitalizations. The variants that showed suggestive association in the discovery phase were evaluated in previously published GWAS results for adults with asthma-related hospitalizations ever with adjustment for age at recruitment, sex, body mass index (BMI), smoking status (never vs. former or current smoking), and the first five principal components of the genotype matrix (49).

Power calculations

GWAS power calculations were carried out in the web-based GAS Power Calculator (50).

Genetic ancestry assessment

All studies assessed genetic ancestry via principal component analysis. Genetic outliers identified by principal components were removed following the criteria described for each study. Although several publications recommend adjusting by the top 10 principal components (51–53), this recommendation is arbitrary and could lead to a loss of power in the absence of population stratification. To evaluate the need to correct for population stratification in each GWAS, the proportion of the genetic variance explained by each principal component was assessed along with variations in the Quantile-Quantile plots and the genomic inflation factor via the step-wise addition of principal components to the GWAS regression model. Principal components that explained a large proportion of the genetic variance and whose addition to the regression model evidenced improvement in control of population stratification via Quantile-Quantile plots and genomic inflation factor closest to 1 were selected for its inclusion in the final GWAS regression model.

**Methylation profiling and quality control**

Genome-wide DNA methylation from whole blood was profiled using the Infinium EPIC BeadChip or the Infinium HumanMethylation450 BeadChip array, as detailed before (54). Bisulfite conversion of 1ug of DNA was performed using the Zymo EZ DNA Methylation Kit (Zymo Research, Irvine, CA), and manufacture protocols were followed for both arrays. We excluded probes with a number of beads<3 or an identification p-value>1x10^-6^ for ≥5% of the samples, samples with low-quality data points for ≥5% of the CpG sites, or total bisulfite intensity less than 3 standard deviations of the sample bisulfite control. Next, we performed "oob" background correction (55), "RELIC" bias correction (56), inter-array ("quantile1") normalization (55), and Regression on Correlated Probes probe-type (RCP) bias adjustment (57). Then, samples with more than 10% of missing probes and probes with missing values in more than 5% of the samples were also removed from further processing and imputation of missing values was performed. Samples with sex discordance according to biological sex and probes on sex chromosomes were also removed. Individuals with mixed genotype distributions on the control single nucleotide polymorphism (SNP) probes were identified with ewastools R package (v1.7) (58) and excluded. In addition, we excluded cross-reactive probes^,^ CpG sites, multimodal probes identified with Enmix (1.22.0) R package (55), probes with a SNP at the single base extension (SBE) or at the CpG site with MAF< 0.01 based on the Illumina manifest file (59). CpG sites were annotated with the Illumina Human Methylation annotation file 10b4 (hg19) and GREAT(60). Beta values, ranging from 0 to 1, were transformed to M-values as log_2_(β/(1-β)).

**Functional assessment of associated SNPs**

DNA methylation quantitative trait loci (meQTL) analyses were conducted using fastQTL (61) for SNPs with MAF≥0.01 and present in at least ten samples, and CpG sites located within 1 Mb of those SNPs. Analyses were performed separately on African Americans, Mexican Americans, and Puerto Ricans, and ethnic-specific results were then meta-analysed with METASOFT (62). Linear regression models were corrected for the asthma exacerbations status, age, sex, the first two genotype principal components, refactor (63) components (6 for GALA II and 7 for SAGE), and methylation batch, when appropriate. SNP-CpG pairs were considered significant at Storey q-value <0.05. *In silico* evidence of functional effects of the variants on gene expression and DNA methylation was assessed using QTLbase (64), the Genotype-Tissue Expression (GTEx) v8 Portal (65), and PhenoScanner v2 (66). Long-distance chromatin interactions were determined using ChiCP (67).

**Association with other traits**

To identify groups of genes previously associated with other traits in the multi-ancestry meta-analysis of GWAS of asthma exacerbations, we used a Gene-Set Enrichment Analysis (GSEA), as implemented in FUMA GWAS (68) via the *GENE2FUNC* algorithm, and queried the GWAS catalog (69). First, SNPs were mapped to the closest gene using the UCSC Table Browser tool (70) for the track UCSC Genes of the human assembly GRCh37/hg19 (February 2009). SNPs with p≤1x10^-4^ were considered since they may be relevant for the trait despite not reaching evidence of suggestive association. The background gene-set considered all types of gene (protein-coding, processed transcripts, etc.,) and the major histocompatibility complex region was excluded from the analysis. A Benjamini-Hochberg-based false discovery rate (FDR) of 5% was used to declare significance.

To estimate the pairwise genome-wide genetic correlations (R_g_) between asthma exacerbations and other traits, we compared our results with publicly-available GWAS summary statistics via LD score regression using LDHub (71). As the majority of GWAS published to date have been conducted in European populations, this analysis was restricted to the results obtained from European-descent individuals of the meta-GWAS to maximize the statistical power. Traits where the LDHub output included the following warnings were excluded from the analysis: “Caution: using these data may yield less robust results due to minor departure of the LD structure” and “Caution: using this data may yield results outside bounds due to relative low Z score of the SNP heritability of the trait”. A Bonferroni-corrected significance threshold of P < 0.05/711 traits = 6.48×10^−5^ was applied.

**Sensitivity analyses**

For each study included in the discovery stage with the required covariate data, sensitivity analyses were conducted following the same procedures as in the main GWAS analysis. To assess the robustness of associations that replicated across stages to the time-dependent probability of occurrence of exacerbations, a stratified analysis was performed separately in those studies were the exacerbation data was collected for the 6 or 12 last months. Moreover, we evaluated the possible confounding effect of body mass index (BMI) categories, obesity, and asthma severity on the genetic association for asthma exacerbations by adding these as covariates in the regression model. For the analysis considering BMI categories, the adult individuals were categorized as follows: a) Underweight (BMI<18.5); b) Normal Weight (BMI ranging 18.5 to 24.9); c) Overweight (BMI ranging 25.0 to 29.9); and d) Obesity (BMI ≥30.0). For individuals under 20 years old, BMI categories were derived from the Centers for Disease Control and Prevention’s BMI-for-age weight status based on percentiles: Underweight (5^th^ BMI percentile); b) Normal Weight (BMI percentile ranging 5^th^ to 85^th^ percentile); c) Overweight (BMI percentile ranging 85^th^ to 95^th^ percentile); and d) Obesity (BMI percentile ≥95^th^ percentile). For the sensitivity analysis assessing the effect of obesity on the genetic association for asthma, only the respective covariate along with those included in the main GWAS analysis were considered in the regression model. For the analysis that evaluated the confounding effect of asthma severity, asthma medication step was used as a proxy of asthma severity in the regression model. Asthma medication step was adapted from British Thoracic Society/Scottish Intercollegiate Guidelines Network guidelines (72); Step 0: No medication; Step 1: SABA as needed: Step 2: SABA as needed plus regular ICS; Step 3: SABA as needed plus regular ICS and LABA; Step 4: SABA as needed plus regular ICS, LABA and LTRA. For COMPASS, the treatment step was adapted from Global Initiative for Asthma (73) and National Asthma Education and Prevention Program guidelines (74). For GALA II and SAGE, the treatment step was adapted from British Thoracic Society/Scottish Intercollegiate Guidelines Network (72) and Global Initiative for Asthma guidelines (73).

In addition, the associations that replicated across stages were evaluated to determine if there were differences by age group. The meta-analysis of the results from studies with data exclusively from children (<18 years old) comprised the ALSPAC, BAMSE, BREATHE, PACMAN, PAGES, PASS, SLOVENIA, and UBIOPRED studies. The meta-analysis of the results from studies with data exclusively from adults (≥18 years old) comprised the GEMAS, MEGAS, the Rotterdam Study, and United Kingdom Biobank studies. For the analysis of adults in GEMAS, 215 individuals with equal or more than 18 years of age were kept.

To address if the association of the main genetic signals could be driven by the underlying asthma susceptibility rather than asthma exacerbations, we searched for association of these variants with asthma using publicly available PheWeb results based on data from the Michigan Genomics Initiative (MGI, MGI freeze2) (75) and the UKB (UKBiobank TOPMed-imputed and UKBiobank HRC-imputed Pheweb results (76,77).

**Literature mining of genetic loci previously associated with asthma exacerbations**

In order to evaluate genetic associations for asthma exacerbations, we conducted a bibliographic search on PubMed for all studies reporting genetic variants significantly associated with asthma exacerbations from 15 December 2018 to June 2021. This updated information was merged with a list of all published studies up to 15 December 2019 available elsewhere (78). For that search, we used combinations of the terms “asthma” AND “exacerbations” OR “hospitalizations” OR “oral corticosteroids” OR “emergency room visits” OR “GWAS” OR “candidate gene” OR “SNP” OR “polymorphism”. After the identification of the publications, we extracted genomic coordinates in the hg19/GRCh37 build and tested/non-tested alleles. Four loci were excluded due to a large overlap in the studies included in the publication reporting the association (8,16,79) and the discovery phase of our study.

**SUPPORTING FIGURES**

**
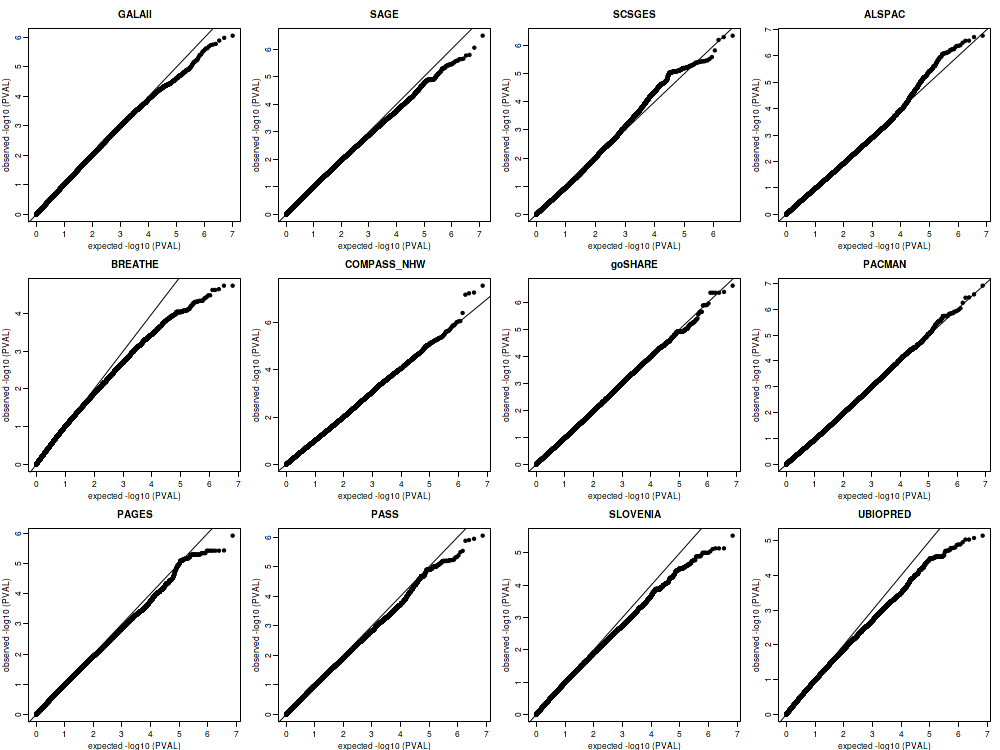
Figure S1. Quantile-quantile plot of association results of severe asthma exacerbations in the studies included in the discovery phase.** Genomic inflation factors for each study were: GALA II: 1.03; SAGE: 1.02; SCSGES: 0.94; ALSPAC: 1.00; BREATHE: 1.07; COMPASS-NHW: 1.04; goSHARE: 0.99; PACMAN: 0.98; PAGES: 1.00; PASS: 1.02; SLOVENIA: 1.02; U-BIOPRED: 1.03.


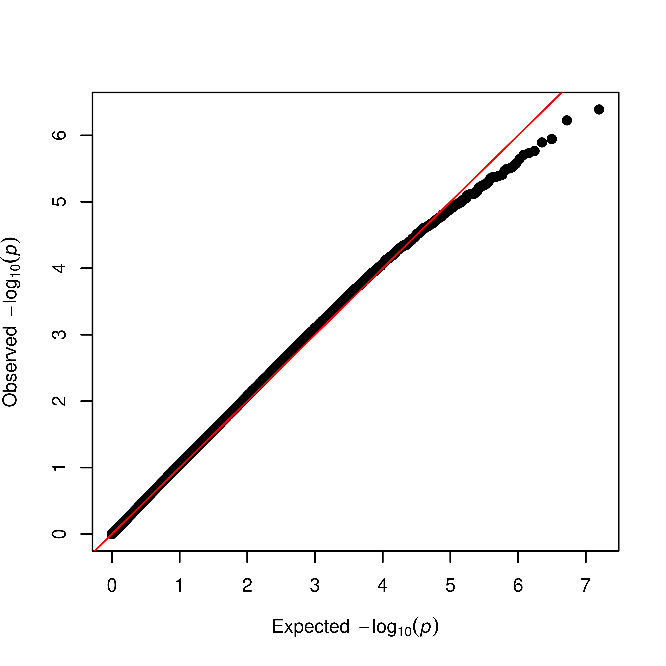


**Figure S2. Quantile-quantile plot of association results of severe asthma exacerbations in the European meta-analysis (genomic inflation factor: 1.07, number of genetic variants: 7,891,404).**


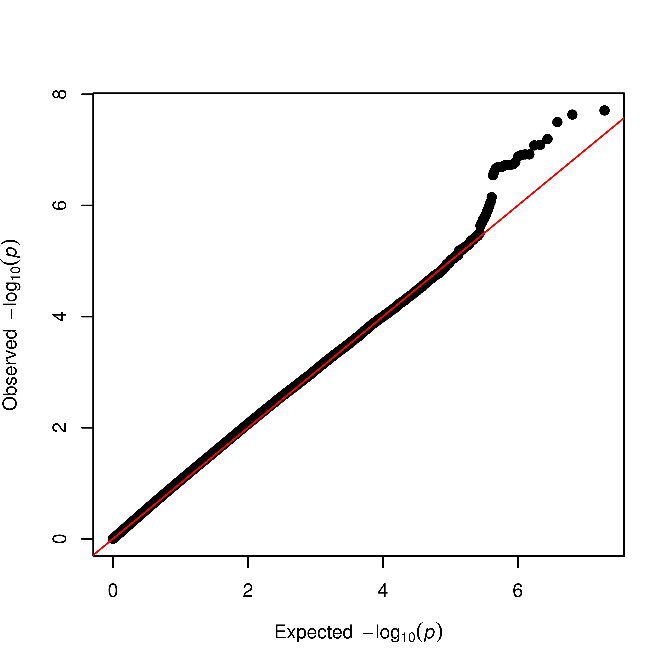


**Figure S3. Quantile-quantile plot of association results of severe asthma exacerbations in the multi-ancestry meta-analysis (genomic inflation factor: 1.11, number of genetic variants: 9,634,748).**

**
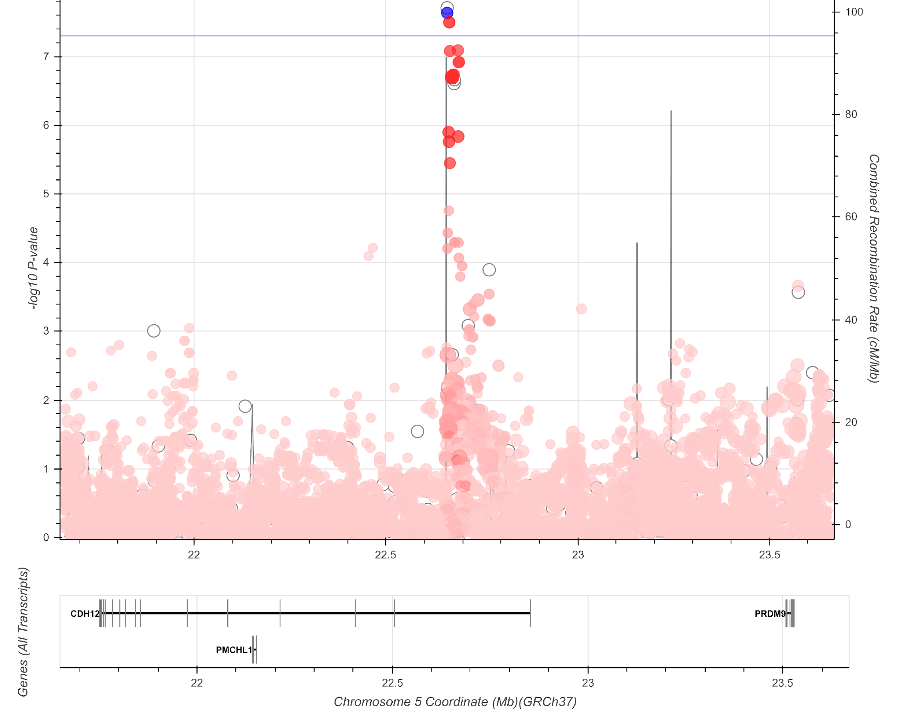
**

**Figure S4. Regional plot of the association results from the multi-ethnic meta-analysis of GWAS of asthma exacerbations in the 5p14.3 locus.** The association results are represented as -log10 p-value on the left y-axis along the chromosomes shown on the x-axis and the recombination rate on the right y-axis. The most significant SNP (rs6884193) in linkage disequilibrium with rs6888198 is shown in blue, and the rest of genetic variants are colored based on the pairwise r^2^ values with rs6884193 (from low in light pink to high in red) according to the data from all the populations from 1000 Genomes Project (80). The genome-wide significance threshold for replication is indicated by the blue line (p=5x10^-8^).

**
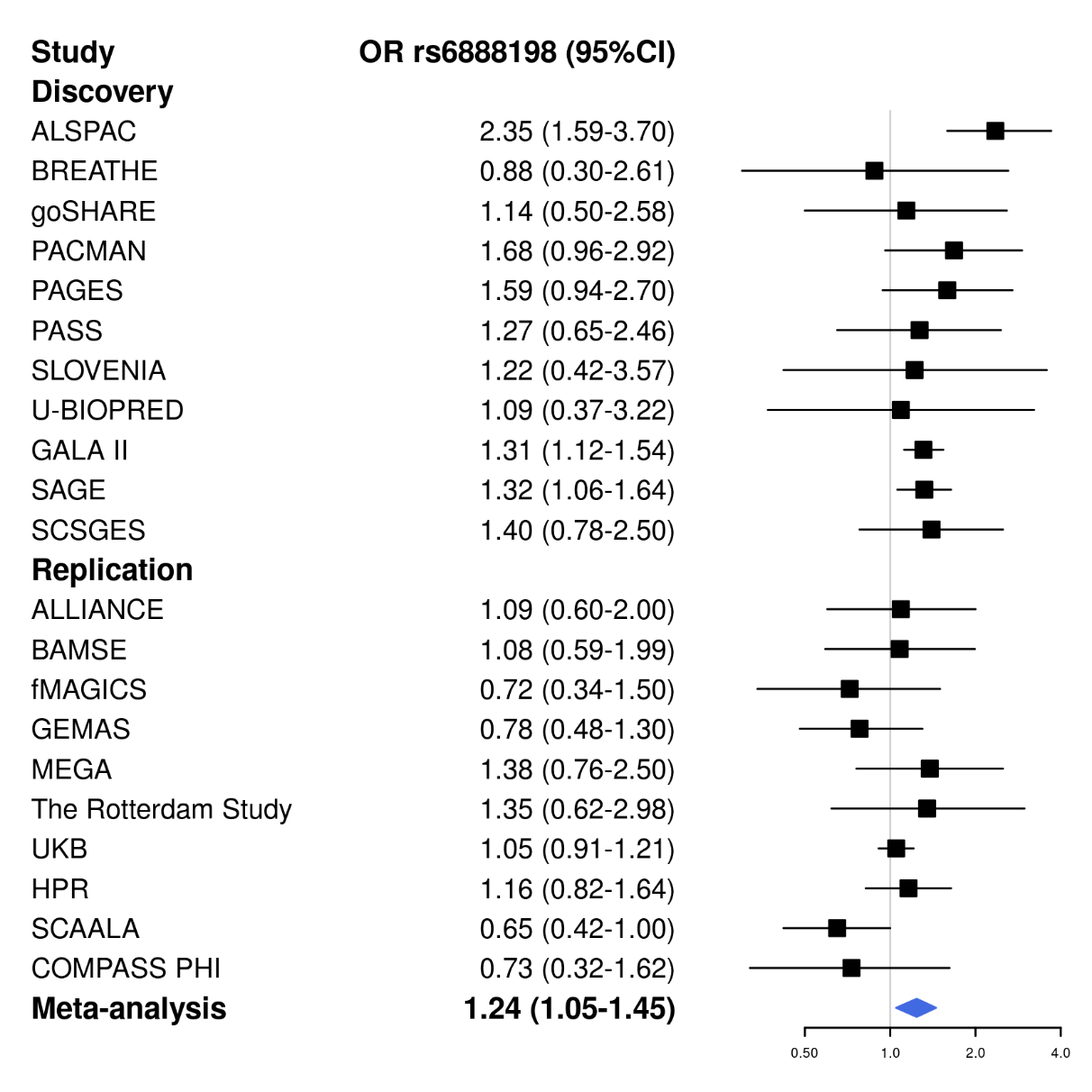
**

**Figure S5. Forest plot of the association results for rs6888198 (*CDH12*) in the meta-analysis of GWAS of asthma exacerbations.**

**
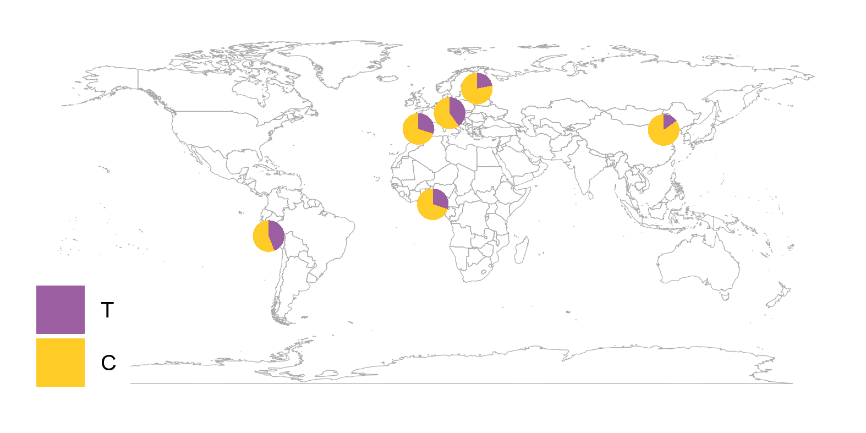
**

**Figure S6. Allele frequency map for rs12091010 SNP in the *EXTL2* locus, according to the Genome Aggregation Database (gnomAD)** (81)**.** Frequency proportions for the effect (T) and non-effect (C) alleles are represented in violet and yellow, respectively. The represented populations include African/African Americans, Ashkenazi Jewish, Latino/Admixed Americans, East Asians, Finnish, and non-Finnish Europeans.


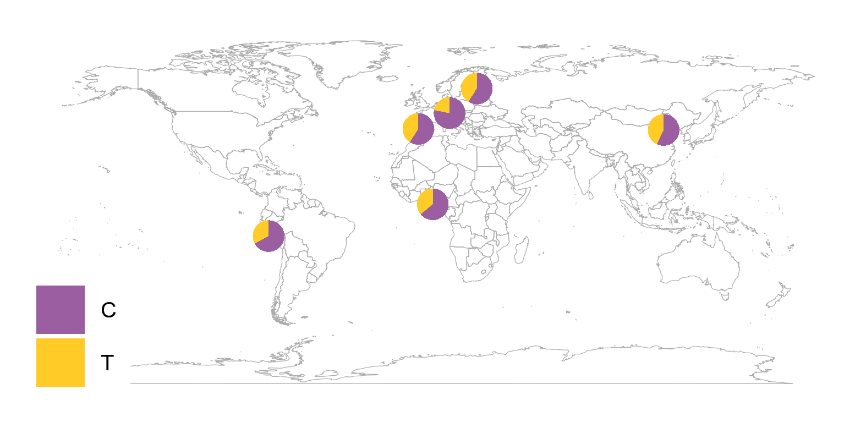


**Figure S7. Allele frequency map for** **rs943126, the most significant SNP in the *PANK1* locus, according to the Genome Aggregation Database (gnomAD)** (81)**.** Frequency proportions for the effect (C) and non-effect (T) alleles are represented in violet and yellow, respectively. The represented populations include African/African Americans, Ashkenazi Jewish, Latino/Admixed Americans, East Asians, Finnish, and non-Finnish Europeans.

**
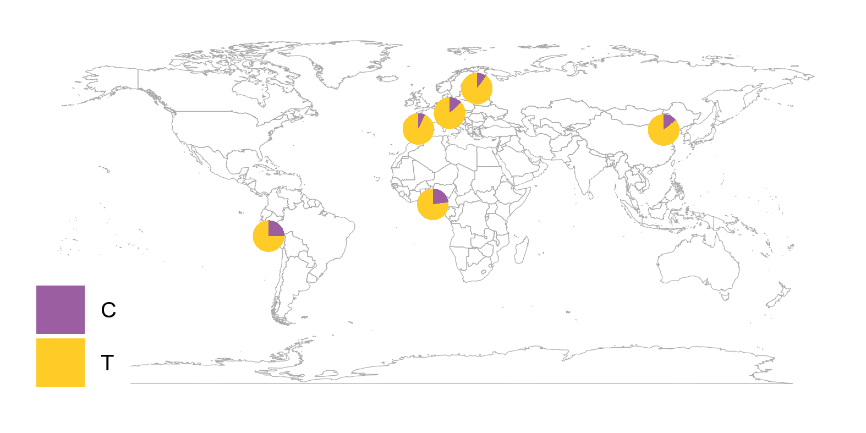
**

**Figure S8. Allele frequency map for rs6888198, the most significant SNP in the *CDH12* locus, according to the Genome Aggregation Database (gnomAD)** (81)**.** Frequency proportions for the effect (C) and non-effect (T) alleles are represented in violet and yellow, respectively. The represented populations include African/African Americans, Ashkenazi Jewish, Latino/Admixed Americans, East Asians, Finnish, and non-Finnish Europeans.

**SUPPORTING REFERENCES**

1. Farzan N, Vijverberg SJ, Andiappan AK, Arianto L, Berce V, Blanca-López N et al. Rationale and design of the multiethnic Pharmacogenomics in Childhood Asthma consortium. *Pharmacogenomics* 2017;**18**:931–943.

2. Fraser A, Macdonald-Wallis C, Tilling K, Boyd A, Golding J, Davey smith G et al. Cohort Profile: the Avon Longitudinal Study of Parents and Children: ALSPAC mothers cohort. *Int J Epidemiol* 2013;**42**:97–110.

3. Boyd A, Golding J, Macleod J, Lawlor DA, Fraser A, Henderson J et al. Cohort Profile: the ’children of the 90s’--the index offspring of the Avon Longitudinal Study of Parents and Children. *Int J Epidemiol* 2013;**42**:111–127.

4. Haag K, Fraser A, Hiller R, Seedat S, Zimmerman A, Halligan SL. The emergence of sex differences in PTSD symptoms across development: evidence from the ALSPAC cohort. *Psychol Med* 2020;**50**:1755–1760.

5. Tavendale R, Macgregor DF, Mukhopadhyay S, Palmer CNAA. A polymorphism controlling ORMDL3 expression is associated with asthma that is poorly controlled by current medications. *J Allergy Clin Immunol* 2008;**121**:860–863.

6. Palmer CNAA, Doney ASFF, Lee SP, Murrie I, Ismail T, Macgregor DF et al. Glutathione S-transferase M1 and P1 genotype, passive smoking, and peak expiratory flow in asthma. *Pediatrics* 2006;**118**:710–716.

7. Palmer CNAA, Lipworth BJ, Lee S, Ismail T, Macgregor DF, Mukhopadhyay S. Arginine-16 beta2 adrenoceptor genotype predisposes to exacerbations in young asthmatics taking regular salmeterol. *Thorax* 2006;**61**:940–944.

8. Hernandez-Pacheco N, Farzan N, Francis B, Karimi L, Repnik K, Vijverberg SJ et al. Genome-wide association study of inhaled corticosteroid response in admixed children with asthma. *Clin Exp Allergy* 2019;**49**:789–798.

9. Kuna P, Peters MJ, Manjra AI, Jorup C, Naya IP, Martínez-Jimenez NE et al. Effect of budesonide/formoterol maintenance and reliever therapy on asthma exacerbations. *Int J Clin Pract* 2007;**61**:725–736.

10. Nishimura KK, Galanter JM, Roth LA, Oh SS, Thakur N, Nguyen EA et al. Early-life air pollution and asthma risk in minority children. The GALA II and SAGE II studies. *Am J Respir Crit Care Med* 2013;**188**:309–318.

11. Borrell LN, Nguyen EA, Roth LA, Oh SS, Tcheurekdjian H, Sen S et al. Childhood obesity and asthma control in the GALA II and SAGE II studies. *Am J Respir Crit Care Med* 2013;**187**:697–702.

12. Thakur N, Oh SS, Nguyen EA, Martin M, Roth LA, Galanter J et al. Socioeconomic status and childhood asthma in urban minority youths. The GALA II and SAGE II studies. *Am J Respir Crit Care Med* 2013;**188**:1202–1209.

13. Herrera-Luis E, Espuela-Ortiz A, Lorenzo-Diaz F, Keys KL, Mak ACY, Eng C et al. Genome-wide association study reveals a novel locus for asthma with severe exacerbations in diverse populations. *Pediatr Allergy Immunol* 2021;**32**:106–115.

14. McKinstry B, Sullivan FM, Vasishta S, Armstrong R, Hanley J, Haughney J et al. Cohort profile: the Scottish Research register SHARE. A register of people interested in research participation linked to NHS data sets. *BMJ Open* 2017;**7**:e013351.

15. Koster ES, Raaijmakers JAAM, Koppelman GH, Postma DS, Van Der Ent CK, Koenderman L et al. Pharmacogenetics of anti-inflammatory treatment in children with asthma: rationale and design of the PACMAN cohort. *Pharmacogenomics* 2009;**10**:1351–1361.

16. Hernandez-Pacheco N, Vijverberg SJ, Herrera-Luis E, Li J, Sio YY, Granell R et al. Genome-wide association study of asthma exacerbations despite inhaled corticosteroid use. *Eur Respir J* 2021;**57**. doi:10.1183/13993003.03388-2020

17. Turner SW, Ayres JG, MacFarlane T V., Mehta A, Mehta G, Palmer CN et al. A methodology to establish a database to study gene environment interactions for childhood asthma. *BMC Med Res Methodol* 2010;**10**:107.

18. Hawcutt DB, Jorgensen AL, Wallin N, Thompson B, Peak M, Lacy D et al. Adrenal responses to a low-dose short synacthen test in children with asthma. *Clin Endocrinol (Oxf)* 2015;**82**:648–656.

19. Hawcutt DB, Francis B, Carr DF, Jorgensen AL, Yin P, Wallin N et al. Susceptibility to corticosteroid-induced adrenal suppression: a genome-wide association study. *Lancet Respir Med* 2018;**6**:442–450.

20. Berce V, Kozmus CEP, Potočnik U. Association among ORMDL3 gene expression, 17q21 polymorphism and response to treatment with inhaled corticosteroids in children with asthma. *Pharmacogenomics J* 2013;**13**:523–529.

21. Andiappan AK, Anantharaman R, Nilkanth PP, Wang DY, Chew FT. Evaluating the transferability of Hapmap SNPs to a Singapore Chinese population. *BMC Genet* 2010;**11**:36.

22. Andiappan AK, Wang DY, Anantharaman R, Parate PN, Suri BK, Low HQ et al. Genome-wide association study for atopy and allergic rhinitis in a Singapore Chinese population. *PLoS One* 2011;**6**:e19719.

23. Sio YY, Matta SA, Ng YT, Chew FT. Epistasis between phenylethanolamine N-methyltransferase and β2-adrenergic receptor influences extracellular epinephrine level and associates with the susceptibility to allergic asthma. *Clin Exp Allergy* 2020;**50**:352–363.

24. Sio YY, Anantharaman R, Lee SQE, Matta SA, Ng YT, Chew FT. The Asthma-associated PER1-like domain-containing protein 1 (PERLD1) Haplotype Influences Soluble Glycosylphosphatidylinositol Anchor Protein (sGPI-AP) Levels in Serum and Immune Cell Proliferation. *Sci Rep* 2020;**10**:715.

25. Andiappan AK, Puan KJ, Lee B, Nardin A, Poidinger M, Connolly J et al. Allergic airway diseases in a tropical urban environment are driven by dominant mono-specific sensitization against house dust mites. *Allergy* 2014;**69**:501–509.

26. Shaw DE, Sousa AR, Fowler SJ, Fleming LJ, Roberts G, Corfield J et al. Clinical and inflammatory characteristics of the European U-BIOPRED adult severe asthma cohort. *Eur Respir J* 2015;**46**:1308–1321.

27. Shrine N, Portelli MA, John C, Soler Artigas M, Bennett N, Hall R et al. Moderate-to-severe asthma in individuals of European ancestry: a genome-wide association study. *Lancet Respir Med* 2019;**7**:20–34.

28. Fuchs O, Bahmer T, Weckmann M, Dittrich A-M, Schaub B, Rösler B et al. The all age asthma cohort (ALLIANCE) - from early beginnings to chronic disease: a longitudinal cohort study. *BMC Pulm Med* 2018;**18**:140.

29. Deutsche Zentren der Gesundheitsforschung. ALLIANCE (All Age Asthma Cohort). https://dzl.de/en/disease-areas/asthma-and-allergy/alliance-all-age-asthma-cohort/

30. Kull I, Melen E, Alm J, Hallberg J, Svartengren M, van Hage M et al. Breast-feeding in relation to asthma, lung function, and sensitization in young schoolchildren. *J Allergy Clin Immunol* 2010;**125**:1013–1019.

31. Melén E, Bergström A, Kull I, Almqvist C, Andersson N, Asarnoj A et al. Male sex is strongly associated with IgE-sensitization to airborne but not food allergens: results up to age 24 years from the BAMSE birth cohort. *Clin Transl Allergy* 2020;**10**:15.

32. Moffatt MF, Gut IG, Demenais F, Strachan DP, Bouzigon E, Heath S et al. A large-scale, consortium-based genomewide association study of asthma. *N Engl J Med* 2010;**363**:1211–1221.

33. Lam M, Awasthi S, Watson HJ, Goldstein J, Panagiotaropoulou G, Trubetskoy V et al. RICOPILI: Rapid Imputation for COnsortias PIpeLIne. *Bioinformatics* 2020;**36**:930–933.

34. McCarthy S, Das S, Kretzschmar W, Delaneau O, Wood AR, Teumer A et al. A reference panel of 64,976 haplotypes for genotype imputation. *Nat Genet* 2016;**48**:1279–1283.

35. Howie BN, Donnelly P, Marchini J. A flexible and accurate genotype imputation method for the next generation of genome-wide association studies. *PLoS Genet* 2009;**5**:e1000529.

36. Nieuwenhuis MA, Siedlinski M, van den Berge M, Granell R, Li X, Niens M et al. Combining genomewide association study and lung eQTL analysis provides evidence for novel genes associated with asthma. *Allergy* 2016;**71**:1712–1720.

37. Pandey RC, Michel S, Schieck M, Binia A, Liang L, Klopp N et al. Polymorphisms in extracellular signal-regulated kinase family influence genetic susceptibility to asthma. *J Allergy Clin Immunol* 2013;**131**:1245–1247.

38. Schieck M, Michel S, Suttner K, Illig T, Zeilinger S, Franke A et al. Genetic variation in TH17 pathway genes, childhood asthma, and total serum IgE levels. *J Allergy Clin Immunol* 2014;**133**:888–891.

39. Perez-Garcia J, Hernández-Pérez JM, González-Pérez R, Sardón O, Martin-Gonzalez E, Espuela-Ortiz A et al. The Genomics and Metagenomics of Asthma Severity (GEMAS) Study: Rationale and Design. *J Pers Med* 2020;**10**:123.

40. R Core Team. R: A language and environment for statistical computing. R Found. Stat. Comput. Vienna, Austria. 2020.

41. Chang CC, Chow CC, Tellier LCAMC, Vattikuti S, Purcell SM, Lee JJ. Second-generation PLINK: rising to the challenge of larger and richer datasets. *Gigascience* 2015;**4**:7.

42. Graffelman J, Moreno V. The mid p-value in exact tests for Hardy-Weinberg equilibrium. *Stat Appl Genet Mol Biol* 2013;**12**. doi:10.1515/sagmb-2012-0039

43. Yan Q, Brehm J, Pino-Yanes M, Forno E, Lin J, Oh SS et al. A meta-analysis of genome-wide association studies of asthma in Puerto Ricans. *Eur Respir J* 2017;**49**:1601505.

44. Yan Q, Forno E, Herrera-Luis E, Pino-Yanes M, Qi C, Rios R et al. A genome-wide association study of severe asthma exacerbations in Latino children and adolescents. *Eur Respir J* 2021;**57**:2002693.

45. Muñoz X, Álvarez-Puebla MJ, Arismendi E, Arochena L, Ausín MDP, Barranco P et al. The MEGA Project: A Study of the Mechanisms Involved in the Genesis and Disease Course of Asthma. Asthma Cohort Creation and Long-Term Follow-Up. *Arch Bronconeumol* 2018;**S0300**-**2896**:30009–7.

46. Ikram MA, Brusselle GGO, Murad SD, van Duijn CM, Franco OH, Goedegebure A et al. The Rotterdam Study: 2018 update on objectives, design and main results. *Eur J Epidemiol* 2017;**32**:807–850.

47. Barreto ML, Cunha SS, Alcântara-Neves N, Carvalho LP, Cruz AA, Stein RT et al. Risk factors and immunological pathways for asthma and other allergic diseases in children: background and methodology of a longitudinal study in a large urban center in Northeastern Brazil (Salvador-SCAALA study). *BMC Pulm Med* 2006;**6**:15.

48. Bycroft C, Freeman C, Petkova D, Band G, Elliott LT, Sharp K et al. The UK Biobank resource with deep phenotyping and genomic data. *Nature* 2018;**562**:203–209.

49. Yan Q, Forno E, Herrera-Luis E, Pino-Yanes M, Yang G, Oh S et al. A genome-wide association study of asthma hospitalizations in adults. *J Allergy Clin Immunol* 2021;**147**:933–940.

50. Johnson JL AG. Gas Power Calculator: Web-Based Power Calculator for Genetic Association Studies. *bioRxiv* 2017.

51. Kang SJ, Larkin EK, Song Y, Barnholtz-Sloan J, Baechle D, Feng T et al. Assessing the impact of global versus local ancestry in association studies. *BMC Proc* 2009;**3 Suppl 7**:S107.

52. Price AL, Zaitlen NA, Reich D, Patterson N. New approaches to population stratification in genome-wide association studies. *Nat Rev Genet* 2010;**11**:459–463.

53. Feng Q, Abraham J, Feng T, Song Y, Elston RC, Zhu X. A method to correct for population structure using a segregation model. *BMC Proc* 2009;**3 Suppl 7**:S104.

54. Herrera-Luis E, Li A, Mak ACY, Perez-Garcia J, Elhawary JR, Oh SS et al. Epigenome-wide association study of lung function in Latino children and youth with asthma. *Clin Epigenetics* 2022;**14**:9.

55. Xu Z, Niu L, Li L, Taylor JA. ENmix: a novel background correction method for Illumina HumanMethylation450 BeadChip. *Nucleic Acids Res* 2016;**44**:e20.

56. Xu Z, Langie SAS, De Boever P, Taylor JA, Niu L. RELIC: a novel dye-bias correction method for Illumina Methylation BeadChip. *BMC Genomics* 2017;**18**:4.

57. Niu L, Xu Z, Taylor JA. RCP: a novel probe design bias correction method for Illumina Methylation BeadChip. *Bioinformatics* 2016;**32**:2659–2663.

58. Heiss JA, Just AC. Identifying mislabeled and contaminated DNA methylation microarray data: an extended quality control toolset with examples from GEO. *Clin Epigenetics* 2018;**10**:73.

59. KD H. IlluminaHumanMethylationEPICanno.ilm10b4.hg19: Annotation for Illumina’s EPIC methylation arrays. R package version 0.6.0. 2016.

60. McLean CY, Bristor D, Hiller M, Clarke SL, Schaar BT, Lowe CB et al. GREAT improves functional interpretation of cis-regulatory regions. *Nat Biotechnol* 2010;**28**:495–501.

61. Ongen H, Buil A, Brown AA, Dermitzakis ET, Delaneau O. Fast and efficient QTL mapper for thousands of molecular phenotypes. *Bioinformatics* 2016;**32**:1479–1485.

62. Han B, Eskin E. Random-effects model aimed at discovering associations in meta-analysis of genome-wide association studies. *Am J Hum Genet* 2011;**88**:586–598.

63. Rahmani E, Zaitlen N, Baran Y, Eng C, Hu D, Galanter J et al. Sparse PCA corrects for cell type heterogeneity in epigenome-wide association studies. *Nat Methods* 2016;**13**:443–445.

64. Zheng Z, Huang D, Wang J, Zhao K, Zhou Y, Guo Z et al. QTLbase: an integrative resource for quantitative trait loci across multiple human molecular phenotypes. *Nucleic Acids Res* 2020;**48**:D983–D991.

65. GTEx Consortium. The GTEx Consortium atlas of genetic regulatory effects across human tissues. *Science* 2020;**369**:1318–1330.

66. Kamat MA, Blackshaw JA, Young R, Surendran P, Burgess S, Danesh J et al. PhenoScanner V2: an expanded tool for searching human genotype-phenotype associations. *Bioinformatics* 2019;**35**:4851–4853.

67. Schofield EC, Carver T, Achuthan P, Freire-Pritchett P, Spivakov M, Todd JA et al. CHiCP: a web-based tool for the integrative and interactive visualization of promoter capture Hi-C datasets. *Bioinformatics* 2016;**32**:2511–2513.

68. Watanabe K, Taskesen E, Van Bochoven A, Posthuma D. Functional mapping and annotation of genetic associations with FUMA. *Nat Commun* 2017;**8**:1826.

69. Buniello A, MacArthur JAL, Cerezo M, Harris LW, Hayhurst J, Malangone C et al. The NHGRI-EBI GWAS Catalog of published genome-wide association studies, targeted arrays and summary statistics 2019. *Nucleic Acids Res* 2019;**47**:D1005–D1012.

70. Karolchik D, Hinrichs AS, Furey TS, Roskin KM, Sugnet CW, Haussler D et al. The UCSC Table Browser data retrieval tool. *Nucleic Acids Res* 2004;**32**:D493-6.

71. Zheng J, Erzurumluoglu AM, Elsworth BL, Kemp JP, Howe L, Haycock PC et al. LD Hub: a centralized database and web interface to perform LD score regression that maximizes the potential of summary level GWAS data for SNP heritability and genetic correlation analysis. *Bioinformatics* 2017;**33**:272–279.

72. British Thoracic Society, Scottish Intercollegiate Guidelines Network. British guideline on the management of asthma. *Thorax* 2014;**69 Suppl 1**:1–192.

73. Global Initiative for Asthma. Global strategy for asthma management and prevention. 2021.http://ginasthma.org/ (accessed 21 Sep2020).

74. National Heart Lung and Blood Institute. Guidelines for the Diagnosis and Management of Asthma (EPR-3). Bethesda 2012

75. Gagliano Taliun SA, VandeHaar P, Boughton AP, Welch RP, Taliun D, Schmidt EM et al. Exploring and visualizing large-scale genetic associations by using PheWeb. *Nat Genet* 2020;**52**:550–552.

76. UKBiobank ICD PheWeb. Analysis of 1403 ICD-based traits using SAIGE. 2021.https://pheweb.org/UKB-SAIGE/

77. UKBiobank TOPMed-imputed PheWeb. Published Online First: 2021.https://pheweb.org/UKB-TOPMed/

78. Herrera-Luis E, Hernandez-Pacheco N, Vijverberg SJ, Flores C, Pino-Yanes M. Role of genomics in asthma exacerbations. *Curr Opin Pulm Med* 2019;**25**:101–112.

79. Slob EMA, Richards LB, Vijverberg SJH, Longo C, Koppelman GH, Pijnenburg MWH et al. Genome-wide association studies of exacerbations in children using long-acting beta2-agonists. *Pediatr Allergy Immunol* 2021;**32**:1197–1207.

80. 1000 Genomes Project Consortium, Auton A, Brooks LD, Durbin RM, Garrison EP, Kang HM et al. A global reference for human genetic variation. *Nature* 2015;**526**:68–74.

81. Karczewski KJ, Francioli LC, Tiao G, Cummings BB, Alföldi J, Wang Q et al. The mutational constraint spectrum quantified from variation in 141,456 humans. *Nature* 2020;**581**:434–443.

**U-BIOPRED Study Group list**

| **U-BIOPRED Supplementary authors** | |
| --- | --- |
| **Name** | **Affiliation** |
| Adcock I M | National Heart and Lung Institute, Imperial College, London, UK; |
| Ahmed H | European Institute for Systems Biology and Medicine, CNRS-ENS-UCBL-INSERM, Lyon, France; |
| Auffray C | European Institute for Systems Biology and Medicine, CNRS-ENS-UCBL-INSERM, Lyon, France; |
| Bakke P | Department of Clinical Science, University of Bergen, Bergen, Norway; |
| Bansal A T | Acclarogen Ltd, St. John’s Innovation Centre, Cambridge, UK; |
| Baribaud F | Janssen R&D, LLC, Spring House, PA, USA |
| Bates S | Respiratory Therapeutic Unit, GSK, London, UK; |
| Bel E H | Academic Medical Centre, University of Amsterdam, Amsterdam, The Netherlands; |
| Bigler J | *Previously Amgen Inc* |
| Bisgaard H | COPSAC, Copenhagen Prospective Studies on Asthma in Childhood, Herlev and Gentofte Hospital,  University of Copenhagen, Copenhagen, Denmark |
| Boedigheimer M J | Amgen Inc.; Thousand Oaks, USA |
| Bønnelykke K | COPSAC, Copenhagen Prospective Studies on Asthma in Childhood, Herlev and Gentofte, Hospital, University of Copenhagen, Copenhagen, Denmark; |
| Brandsma J | University of Southampton, Southampton, UK |
| Brinkman P | Academic Medical Centre, University of Amsterdam, Amsterdam, The Netherlands; |
| Bucchioni E | Chiesi Pharmaceuticals SPA, Parma, Italy |
| Burg D | Centre for Proteomic Research, Institute for Life Sciences, University of Southampton, Southampton, UK |
| Bush A | National Heart and Lung Institute, Imperial College, London, UK; Royal Brompton and Harefield NHS trust, UK |
| Caruso M | Dept. Clinical and Experimental Medicine, University of Catania, Catania, Italy; |
| Chaiboonchoe A | European Institute for Systems Biology and Medicine, CNRS-ENS-UCBL-INSERM, Lyon, France; |
| Chanez P | Assistance publique des Hôpitaux de Marseille - Clinique des bronches, allergies et sommeil, Aix Marseille Université, Marseille, France |
| Chung K F | National Heart and Lung Institute, Imperial College, London, UK; |
| Compton C H | Respiratory Therapeutic Unit, GSK, London, UK |
| Corfield J | Areteva R&D, Nottingham, UK; |
| Cunoosamy D | Sanofi, Cambridge, USA |
| D’Amico A | University of Rome ‘Tor Vergata’, Rome Italy; |
| Dahlén B | Karolinska University Hospital & Centre for Allergy Research, Karolinska Institutet, Stockholm, Sweden |
| Dahlén S E | Centre for Allergy Research, Karolinska Institutet, Stockholm, Sweden |
| De Meulder B | European Institute for Systems Biology and Medicine, CNRS-ENS-UCBL-INSERM, Lyon, France; |
| Djukanovic R | NIHR Southampton Respiratory Biomedical Research Unit and Clinical and Experimental Sciences, Southampton, UK; |
| Erpenbeck V J | Translational Medicine, Respiratory Profiling, Novartis Institutes for Biomedical Research, Basel, Switzerland; |
| Erzen D | Boehringer Ingelheim Pharma GmbH & Co. KG; Biberach, Germany |
| Fichtner K | Boehringer Ingelheim Pharma GmbH & Co. KG; Biberach, Germany |
| Fleming L J | National Heart and Lung Institute, Imperial College, London, UK; Royal Brompton and Harefield NHS trust, UK |
| Formaggio E | *Previously CROMSOURCE, Verona Italy* |
| Fowler S J | Division of infection, immunity and respiratory medicine, School of biological sciences, University of Manchester, Manchester University NHS Foundation Trust, Manchester Academic Health Science Centre, Manchester, UK |
| Frey U | University Children’s Hospital, Basel, Switzerland; |
| Gahlemann M | Boehringer Ingelheim (Schweiz) GmbH,Basel, Switzerland; |
| Geiser T | Department of Respiratory Medicine, University Hospital Bern, Switzerland; |
| Goss V | NIHR Respiratory Biomedical Research Unit, University Hospital Southampton NHS Foundation Trust, Integrative Physiology and Critical Illness Group, Clinical and Experimental Sciences, Sir Henry Wellcome Laboratories, Faculty of Medicine, University of Southampton, Southampton, UK; |
| Guo Y | Data Science Institute, Imperial College, London, UK; |
| Hashimoto S | Academic Medical Centre, University of Amsterdam, Amsterdam, The Netherlands; |
| Haughney J | International Primary Care Respiratory Group, Aberdeen, Scotland; |
| Hedlin G | Dept. Women’s and Children’s Health & Centre for Allergy Research, Karolinska Institutet, Stockholm, Sweden; |
| Hekking P W | Academic Medical Centre, University of Amsterdam, Amsterdam, The Netherlands; |
| Higenbottam T | Allergy Therapeutics, West Sussex, UK; |
| Hohlfeld J M | Fraunhofer Institute for Toxicology and Experimental Medicine, Hannover, Germany |
| Holweg C | Respiratory and Allergy Diseases, Genentech, San Francisco, USA |
| Horváth I | Semmelweis University, Budapest, Hungary |
| Howarth P | NIHR Southampton Respiratory Biomedical Research Unit, Clinical and Experimental Sciences and Human Development and Health, Southampton, UK |
| James A J | Centre for Allergy Research, Karolinska Institutet, Stockholm, Sweden; |
| Knowles R G | Knowles Consulting Ltd, Stevenage. UK; |
| Knox A J | Respiratory Research Unit, University of Nottingham, Nottingham, UK; |
| Krug N | Fraunhofer Institute for Toxicology and Experimental Medicine, Hannover, Germany; |
| Lefaudeux D | European Institute for Systems Biology and Medicine, CNRS-ENS-UCBL-INSERM, Lyon, France; |
| Loza M J | Janssen R&D, LLC, Spring House, PA, USA |
| Lutter R | Academic Medical Centre, University of Amsterdam, Amsterdam, The Netherlands; |
| Manta A | Roche Diagnostics GmbH, Mannheim, Germany |
| Masefield S | European Lung Foundation, Sheffield, UK; |
| Matthews J G | Respiratory and Allergy Diseases, Genentech, San Francisco, USA; |
| Mazein A | European Institute for Systems Biology and Medicine, CNRS-ENS-UCBL-INSERM, Lyon, France |
| Meiser A | Data Science Institute, Imperial College, London, UK |
| Middelveld R J M | Centre for Allergy Research, Karolinska Institutet, Stockholm, Sweden |
| Miralpeix M | Almirall, Barcelona, Spain; |
| Montuschi P | Università Cattolica del Sacro Cuore, Milan, Italy; |
| Mores N | Università Cattolica del Sacro Cuore, Milan, Italy; |
| Murray C S | Division of infection, immunity and respiratory medicine, School of biological sciences, University of Manchester, Manchester University NHS Foundation Trust, and Manchester Academic Health Science Centre, Manchester, United Kingdom |
| Musial J | Dept. of Medicine, Jagiellonian University Medical College, Krakow, Poland |
| Myles D | Respiratory Therapeutic Unit, GSK, London, UK; |
| Pahus L | Assistance publique des Hôpitaux de Marseille, Clinique des bronches, allergies et sommeil  Espace Éthique Méditerranéen, Aix-Marseille Université, Marseille, France; |
| Pandis I | Data Science Institute, Imperial College, London, UK |
| Pavlidis S | National Heart and Lung Institute, Imperial College, London, UK |
| Postle A | University of Southampton, UK |
| Powel P | European Lung Foundation, Sheffield, UK; |
| Praticò G | CROMSOURCE, Verona, Italy |
| Puig Valls M | CROMSOURCE, Barcelona, Spain |
| Rao N | Janssen R&D, LLC, Spring House, PA, USA |
| Riley J | Respiratory Therapeutic Unit, GSK, London, UK; |
| Roberts A | Asthma UK, London, UK; |
| Roberts G | NIHR Southampton Respiratory Biomedical Research Unit, Clinical and Experimental Sciences and Human Development and Health, Southampton, UK; |
| Rowe A | Janssen R&D, UK; |
| Sandström T | Dept of Public Health and Clinical Medicine, Umeå University, Umeå, Sweden; |
| Schofield J P R | Centre for Proteomic Research, Institute for Life Sciences, University of Southampton, Southampton, UK |
| Seibold W | Boehringer Ingelheim Pharma GmbH, Biberach, Germany |
| Selby A | NIHR Southampton Respiratory Biomedical Research Unit, Clinical and Experimental Sciences and Human Development and Health, Southampton, UK; |
| Shaw D E | Respiratory Research Unit, University of Nottingham, UK; |
| Sigmund R | Boehringer Ingelheim Pharma GmbH & Co. KG; Biberach, Germany |
| Singer F | Pediatric Respiratory Medicine, Department of Pediatrics, Inselspital, Bern University Hospital, University of Bern, Bern, Switzerland. |
| Skipp P J | Centre for Proteomic Research, Institute for Life Sciences, University of Southampton, Southampton, UK |
| Smicker M | Sanofi, Cambridge, USA |
| Sousa A R | Respiratory Therapeutic Unit, GSK, London, UK; |
| Sterk P J | Academic Medical Centre, University of Amsterdam, Amsterdam, The Netherlands; |
| Sun K | Data Science Institute, Imperial College, London, UK |
| Thornton B | MSD, USA |
| Uddin M | AstraZeneca BioPharmaceuticals R&D, Gothenburg, Sweden |
| van Aalderen W M | Academic Medical Centre, University of Amsterdam, Amsterdam, The Netherlands; |
| Vestbo J | Centre for Respiratory Medicine and Allergy, Institute of Inflammation and Repair, University of Manchester and University Hospital of South Manchester, Manchester Academic Health Sciences Centre, Manchester, United Kingdom |
| Vissing N H | COPSAC, Copenhagen Prospective Studies on Asthma in Childhood, Herlev and Gentofte Hospital, and University of Copenhagen, Copenhagen, Denmark; |
| Wagener A H | Academic Medical Center Amsterdam, Amsterdam, The Netherlands |
| Wagers S S | BioSci Consulting, Maasmechelen, Belgium |
| Weiszhart Z | Semmelweis University, Budapest, Hungary; |
| Wheelock A | Respiratory Medicine Unit, Department of Medicine Solna and Center for Molecular Medicine, Karlinska Institutet, Stockholm, Sweden; and Department of Respiratory Medicine and Allergy, Karolinska University Hospital Solna, Stockholm, Sweden; |
| Wheelock C E | Centre for Allergy Research, Karolinska Institutet, Stockholm, Sweden; |
| Wilson S J | Histochemistry Research Unit, Faculty of Medicine, University of Southampton, Southampton, UK; |

**Contributors**

| Aliprantis Antonios, Merck Research Laboratories, Boston, USA; |
| --- |
| Allen David, North West Severe Asthma Network, Pennine Acute Hospital NHS Trust, UK |
| Alving Kjell, Dept Women’s & Children’s Health, Uppsala University, Uppsala, Sweden |
| Badorrek P, Fraunhofer ITEM; Hannover, Germany |
| Balgoma David, Centre for Allergy Research, Karolinska Institutet, Stockholm, Sweden |
| Ballereau S, European institute for Systems Biology and Medicine, University of Lyon, France |
| Barber Clair, NIHR Southampton Respiratory Biomedical Research Unit and Clinical and Experimental Sciences, Southampton, UK; |
| Batuwitage Manohara Kanangana, Data Science Institute, Imperial College, London, UK |
| Bautmans An, MSD, Brussels, Belgium |
| Bedding A, Roche Diagnostics GmbH, Mannheim, Germany |
| Behndig AF, Umeå University, Umea, Sweden |
| Beleta Jorge, Almirall S.A., Barcelona, Spain; |
| Berglind A, MSD, Brussels, Belgium |
| Berton A, AstraZeneca BioPharmaceuticals R&D, Gothenburg, Sweden |
| Bochenek Grazyna, II Department of Internal Medicine, Jagiellonian University Medical College, Krakow, Poland; |
| Braun Armin, Fraunhofer Institute for Toxicology and Experimental Medicine, Hannover, Germany; |
| Campagna D, Department of Clinical and Experimental Medicine, University of Catania, Catania, Italy; |
| *Carayannopoulos Leon,* *Previously at: MSD, USA;* |
| Casaulta C, University Children’s Hospital of Bern, Switzerland |
| Chaleckis Romanas, Centre of Allergy Research, Karolinska Institutet, Stockholm, Sweden |
| Davison Timothy Janssen R&D, LLC, Spring House, PA, USA |
| De Alba Jorge, Almirall S.A., Barcelona, Spain; |
| De Lepeleire Inge, MSD, Brussels, BE |
| Dekker Tamara, Academic Medical Centre, University of Amsterdam, Amsterdam, The Netherlands; |
| Delin Ingrid, Centre for Allergy Research, Karolinska Institutet, Stockholm, Sweden |
| Dennison P, NIHR Southampton Respiratory Biomedical Research Unit, Clinical and Experimental Sciences, NIHR-Wellcome Trust Clinical Research Facility, Faculty of Medicine, University of Southampton, Southampton, UK; |
| Dijkhuis Annemiek, Academic Medical Centre, University of Amsterdam, Amsterdam, The Netherlands; |
| Dodson Paul, AstraZeneca BioPharmaceuticals R&D, Gothenburg, Sweden |
| Draper Aleksandra, BioSci Consulting, Maasmechelen, Belgium; |
| Dyson K, CROMSOURCE; Stirling, UK |
| Edwards Jessica, Asthma UK, London, UK; |
| El Hadjam L, European Institute for Systems Biology and Medicine, University of Lyon |
| Emma Rosalia, Department of Clinical and Experimental Medicine, University of Catania, Catania, Italy; |
| Ericsson Magnus, Karolinska University Hospital, Stockholm, Sweden |
| Faulenbach C, Fraunhofer ITEM; Hannover, Germany |
| Flood Breda, European Federation of Allergy and Airways Diseases Patient’s Associations, Brussels, Belgium |
| Fowler Stephan S, Centre for respiratory medicine and allergy, Institute of Inflammation and repair, University Hospital of South Manchester, NHS Foundation Trust, Manchester, UK |
| Galffy G, Semmelweis University, Budapest, Hungary; |
| Gallart Hector, Centre for Allergy Research, Karolinska Institutet, Stockholm, Sweden |
| Garissi D, Global Head Clinical Research Division, CROMSOURCE, Italy |
| Gent J, Royal Brompton and Harefield NHS Foundation Trust, London, UK; |
| Gerhardsson de Verdier M, AstraZeneca BioPharmaceuticals R&D, Gothenburg, Sweden |
| Gibeon D, National Heart and Lung Institute, Imperial College, London, UK; |
| Gomez Cristina, Centre for Allergy Research, Karolinska Institutet, Stockholm, Sweden |
| Gove Kerry, NIHR Southampton Respiratory Biomedical Research Unit and Clinical and Experimental Sciences, Southampton, UK; |
| Gozzard Neil, UCB, Slough, UK; |
| Guillmant-Farry E, Royal Brompton Hospital, London, UK |
| Henriksson E, Karolinska University Hospital & Karolinska Institutet, Stockholm, Sweden |
| Hewitt Lorraine, NIHR Southampton Respiratory Biomedical Research Unit, Southampton, UK |
| Hoda U, Imperial College, London, UK |
| Hu Richard, Amgen Inc. Thousand Oaks, USA |
| Hu Sile, National Heart and Lung Institute, Imperial College, London, UK; |
| Hu X, Amgen Inc.; Thousand Oaks, USA |
| Jeyasingham E, UK Clinical Operations, GSK, Stockley Park, UK |
| Johnson K, Centre for respiratory medicine and allergy, Institute of Inflammation and repair, University Hospital of South Manchester, NHS Foundation Trust, Manchester, UK |
| Jullian N, European Institute for Systems Biology and Medicine, University of Lyon |
| Kamphuis Juliette, Longfonds, Amersfoort, The Netherlands; |
| Kennington Erika J., Asthma UK, London, UK; |
| Kerry Dyson, CromSource, Stirling, UK; |
| Kerry G, Centre for respiratory medicine and allergy, Institute of Inflammation and repair, University Hospital of South Manchester, NHS Foundation Trust, Manchester, UK |
| Klüglich M, Boehringer Ingelheim Pharma GmbH & Co. KG; Biberach, Germany |
| Knobel Hugo, Philips Research Laboratories, Eindhoven, The Netherlands; |
| Kolmert Johan, Centre for Allergy Research, Karolinska Institutet, Stockholm, Sweden |
| Konradsen J R, Dept. Women’s and Children’s Health & Centre for Allergy Research, Karolinska Institutet, Stockholm, Sweden |
| Kots Maxim, Chiesi Pharmaceuticals, SPA, Parma, Italy; |
| Kretsos Kosmas, UCB, Slough, UK |
| Krueger L, University Children's Hospital Bern, Switzerland |
| Kuo Scott, National Heart and Lung Institute, Imperial College, London, UK; |
| Kupczyk Maciej, Centre for Allergy Research, Karolinska Institutet, Stockholm, Sweden |
| Lambrecht Bart, University of Gent, Gent, Belgium; |
| Lantz A-S, Karolinska University Hospital & Centre for Allergy Research, Karolinska Institutet, Stockholm, Sweden |
| Larminie Christopher, GSK, London, UK |
| Larsson L X, AstraZeneca BioPharmaceuticals R&D, Gothenburg, Sweden |
| Latzin P, University Children’s Hospital of Bern, Bern, Switzerland |
| Lazarinis N, Karolinska University Hospital & Karolinska Institutet, Stockholm, Sweden |
| Lemonnier N, European Institute for Systems Biology and Medicine, CNRS-ENS-UCBL-INSERM, Lyon, France |
| Lone-Latif Saeeda, Academic Medical Centre, University of Amsterdam, Amsterdam, The Netherlands; |
| Lowe L A, Centre for respiratory medicine and allergy, Institute of Inflammation and repair, University Hospital of South Manchester, NHS Foundation Trust, Manchester, UK |
| Manta Alexander, Roche Diagnostics GmbH, Mannheim, Germany |
| Marouzet Lisa, NIHR Southampton Respiratory Biomedical Research Unit, Southampton, UK |
| Martin Jane, NIHR Southampton Respiratory Biomedical Research Unit, Southampton, UK |
| Mathon Caroline, Centre of Allergy Research, Karolinska Institutet, Stockholm, Sweden |
| McEvoy L, University Hospital, Department of Pulmonary Medicine, Bern, Switzerland |
| Meah Sally, National Heart and Lung Institute, Imperial College, London, UK; |
| Menzies-Gow A, Royal Brompton and Harefield NHS Foundation Trust, London, UK; |
| *Metcalf Leanne, Previously at: Asthma UK, London, UK;* |
| Mikus Maria, Science for Life Laboratory & The Royal Institute of Technology, Stockholm, Sweden; |
| Monk Philip, Synairgen Research Ltd, Southampton, UK; |
| Mumby Sharon, National Heart and Lung Institute, Imperial College, London, UK |
| Naz Shama, Centre for Allergy Research, Karolinska Institutet, Stockholm, Sweden |
| Nething K, Boehringer Ingelheim Pharma GmbH & Co. KG; Biberach, Germany |
| Nicholas Ben, University of Southampton, Southampton, UK |
| Nihlén U, *Previously* AstraZeneca BioPharmaceuticals R&D, Gothenburg, Sweden |
| Nilsson Peter, Science for Life Laboratory & The Royal Institute of Technology, Stockholm, Sweden; |
| Niven R, North West Severe Asthma Network, University Hospital South Manchester, UK |
| Nordlund B, Dept. Women’s and Children’s Health & Centre for Allergy Research, Karolinska Institutet, Stockholm, Sweden |
| Nsubuga S, Royal Brompton Hospital, London, UK |
| Pacino Antonio, Lega Italiano Anti Fumo, Catania, Italy; |
| Palkonen Susanna, European Federation of Allergy and Airways Diseases Patient’s Associations, Brussels, Belgium. |
| Pellet J, European Institute for Systems Biology and Medicine, CNRS-ENS-UCBL-INSERM, Lyon, France |
| Pennazza Giorgio, Unit of Electronics for Sensor Systems, Department of Engineering, Campus Bio-Medico University of Rome, Rome, Italy |
| Petrén Anne, Centre for Allergy Research, Karolinska Institutet, Stockholm, Sweden |
| Pink Sandy, NIHR Southampton Respiratory Biomedical Research Unit, Southampton, UK |
| Pison C, European Institute for Systems Biology and Medicine, CNRS-ENS-UCBL-INSERM, Lyon, France |
| *Rahman-Amin Malayka, Previously at: Asthma UK, London, UK;* |
| Ravanetti Lara, Academic Medical Centre, University of Amsterdam, Amsterdam, The Netherlands; |
| Ray Emma, NIHR Southampton Respiratory Biomedical Research Unit, Southampton, UK |
| Reinke Stacey, Centre for Allergy Research, Karolinska Institutet, Stockholm, Sweden |
| *Reynolds Leanne, Previously at: Asthma UK, London, UK;* |
| Riemann K, Boehringer Ingelheim Pharma GmbH & Co. KG; Biberach, Germany |
| Robberechts Martine, MSD, Brussels, Belgium |
| Rocha J P, Royal Brompton and Harefield NHS Foundation Trust |
| Rossios C, National Heart and Lung Institute, Imperial College, London, UK; |
| Russell Kirsty, National Heart and Lung Institute, Imperial College, London, UK; |
| Rutgers Michael, Longfonds, Amersfoort, The Netherlands; |
| Santini G, Università Cattolica del Sacro Cuore, Milan, Italy; |
| Santonico Marco, Unit of Electronics for Sensor Systems, Department of Engineering, Campus Bio-Medico University of Rome, Rome, Italy |
| Saqi M, European Institute for Systems Biology and Medicine, CNRS-ENS-UCBL-INSERM, Lyon, France |
| Schoelch Corinna, Boehringer Ingelheim Pharma GmbH & Co. KG, Biberach, Germany |
| Scott S, North West Severe Asthma Network, Countess of Chester Hospital, UK |
| Sehgal N, North West Severe Asthma Network; Pennine Acute Hospital NHS Trust |
| Sjödin Marcus, Centre for Allergy Research, Karolinska Institutet, Stockholm, Sweden |
| Smids Barbara, Academic Medical Centre, University of Amsterdam, Amsterdam, The Netherlands; |
| Smith Caroline, NIHR Southampton Respiratory Biomedical Research Unit, Southampton, UK |
| Smith Jessica, Asthma UK, London, UK; |
| Smith Katherine M., University of Nottingham, UK; |
| Söderman P, Dept. Women’s and Children’s Health, Karolinska Institutet, Stockholm, Sweden |
| Sogbesan A, Royal Brompton and Harefield NHS Foundation Trust, London, UK; |
| Spycher F, University Hospital Department of Pulmonary Medicine, Bern, Switzerland |
| Staykova Doroteya, University of Southampton, Southampton, UK |
| Stokholm J, University of Copenhagen and Danish Pediatric Asthma Centre Denmark |
| Strandberg K, Karolinska University Hospital & Karolinska Institutet, Stockholm, Sweden |
| Sunther M, Centre for respiratory medicine and allergy, Institute of Inflammation and repair, University Hospital of South Manchester, NHS Foundation Trust, Manchester, UK |
| Szentkereszty M, Semmelweis University, Budapest, Hungary; |
| Tamasi L, Semmelweis University, Budapest, Hungary; |
| Tariq K, NIHR Southampton Respiratory Biomedical Research Unit, Clinical and Experimental Sciences, NIHR-Wellcome Trust Clinical Research Facility, Faculty of Medicine, University of Southampton, Southampton, UK; |
| Thörngren John-Olof, Karolinska University Hospital, Stockholm, Sweden |
| Thorsen Jonathan, COPSAC, Copenhagen Prospective Studies on Asthma in Childhood, Herlev and Gentofte  Hospital, University of Copenhagen, Copenhagen, Denmark; |
| Valente S, Università Cattolica del Sacro Cuore, Milan, Italy; |
| van de Pol Marianne, Academic Medical Centre, University of Amsterdam, Amsterdam ,The Netherlands; |
| van Drunen C M, Academic Medical Centre, University of Amsterdam, Amsterdam, The Netherlands; |
| Van Eyll Jonathan, UCB, Slough, UK |
| *Versnel Jenny, Previously at: Asthma UK, London, UK;* |
| Vink Anton, Philips Research Laboratories, Eindhoven, The Netherlands; |
| von Garnier C, University Hospital Bern, Switzerland; |
| Vyas A, North west Severe Asthma Network, Lancashire Teaching Hospitals NHS Trust, UK |
| Wald Frans, Boehringer Ingelheim Pharma GmbH & Co. KG, Biberach, Germany |
| Walker Samantha, Asthma UK, London, UK; |
| Ward Jonathan, Histochemistry Research Unit, Faculty of Medicine, University of Southampton, Southampton, UK; |
| Wetzel Kristiane, Boehringer Ingelheim Pharma GmbH, Biberach, Germany |
| Wiegman Coen, National Heart and Lung Institute, Imperial College, London, UK; |
| Williams Siân, International Primary Care Respiratory Group, Aberdeen, Scotland; |
| Yang Xian, Data Science Institute, Imperial College, London, UK |
| Yeyasingham Elizabeth, UK Clinical Operations, GSK, Stockley Park, UK; |
| Yu W, Amgen Inc.; Thousand Oaks, USA |
| Zetterquist W, Dept. Women’s and Children’s Health & Centre for Allergy Research, Karolinska Institutet, Stockholm, Sweden |
| Zolkipli Z, NIHR Southampton Respiratory Biomedical Research Unit, Clinical and Experimental Sciences and Human Development and Health, Southampton, UK; |
| Zwinderman A H, Academic Medical Centre, University of Amsterdam, The Netherlands; |

| **Partner organisations** | |
| --- | --- |
| Novartis Pharma AG | University of Southampton, Southampton, UK |
| Academic Medical Centre, University of Amsterdam, Amsterdam, The Netherlands | Imperial College London, London, UK |
| University of Catania, Catania, Italy | University of Rome ‘Tor Vergata’, Rome, Italy |
| Hvidore Hospital, Hvidore, Denmark | Jagiellonian Univ. Medi.College, Krakow, Poland |
| University Hospital, Inselspital, Bern, Switzerland | Semmelweis University, Budapest, Hungary |
| University of Manchester, Manchester, UK | Université d’Aix-Marseille, Marseille, France |
| Fraunhofer Institute, Hannover, Germany | University Hospital, Umea, Sweden |
| Ghent University, Ghent, Belgium | Ctr. Nat. Recherche Scientifique, Lyon, France |
| Università Cattolica del Sacro Cuore, Rome, Italy | University Hospital, Copenhagen, Denmark |
| Karolinska Institutet, Stockholm, Sweden | Nottingham University Hospital, Nottingham, UK |
| University of Bergen, Bergen, Norway | Netherlands Asthma Foundation, Leusden, NL |
| European Lung Foundation, Sheffield, UK | Asthma UK, London, UK |
| European. Fed. of Allergy and Airways Diseases Patients’ Associations, Brussels, Belgium | Lega Italiano Anti Fumo, Catania, Italy |
| International Primary Care Respiratory Group, Aberdeen, Scotland | Philips Research Laboratories, Eindhoven, NL |
| Synairgen Research Ltd, Southampton, UK | Aerocrine AB, Stockholm, Sweden |
| BioSci Consulting, Maasmechelen, Belgium | Almirall |
| AstraZeneca BioPharmaceuticals R&D | Boehringer Ingelheim |
| Chiesi | GlaxoSmithKline |
| Roche | UCB |
| Janssen Biologics BV | Amgen NV |

| **MEMBERS OF THE ETHICS BOARD** | | | |
| --- | --- | --- | --- |
| **Name** | **Task** | **Affiliation** | **e-mail** |
| Jan-Bas Prins | Biomedical research | LUMC/the Netherlands | J.B.Prins@lumc.nl |
| Martina Gahlemann | Clinical care | BI/Germany | Martina.Gahlemann@boehringer-ingelheim.com |
| Luigi Visintin | Legal affairs | LIAF/Italy | visintin@inrete.it |
| Hazel Evans | Paediatric care | Southampton/UK | hazel.evans@uhs.nhs.uk |
| Martine Puhl | Patient representation (co chair) | NAF/ the Netherlands | martine@puhl.nl |
| Lina Buzermaniene | Patient representation | EFA/Lithuania | lina.buzermaniene@pavb.lt |
| Val Hudson | Patient representation | Asthma UK | hudsonval7@gmail.com |
| Laura Bond | Patient representation | Asthma UK | lvbond22@googlemail.com |
| Pim de Boer | Patient representation and pathobiology | IND | deboer.pim@hetnet.nl |
| Guy Widdershoven | Research ethics | VUMC/the Netherlands | g.widdershoven@vumc.nl |
| Ralf Sigmund | Research methodology and biostatistics | BI/Germany | ralf.sigmund@boehringer-ingelheim.com |

| **THE PATIENT INPUT PLATFORM** | |
| --- | --- |
| **Name** | **Country** |
| Amanda Roberts | UK |
| David Supple (chair) | UK |
| Dominique Hamerlijnck | The Netherlands |
| Jenny Negus | UK |
| Juliёtte Kamphuis | The Netherlands |
| Lehanne Sergison | UK |
| Luigi Visintin | Italy |
| Pim de Boer (co-chair) | The Netherlands |
| Susanne Onstein | The Netherlands |

| **MEMBERS OF THE SAFETY MONITORING BOARD** | |
| --- | --- |
| **Name** | **Task** |
| William MacNee | Clinical care |
| Renato Bernardini | Clinical pharmacology |
| Louis Bont | Paediatric care and infectious diseases |
| Per-Ake Wecksell | Patient representation |
| Pim de Boer | Patient representation and pathobiology (chair) |
| Martina Gahlemann | Patient safety advice and clinical care (co-chair) |
| Ralf Sigmund | Bio-informatician |
